# Expression of HER-2 affects patient survival and paclitaxel sensitivity in endometrial cancer

**DOI:** 10.1038/sj.bjc.6605805

**Published:** 2010-07-27

**Authors:** N Mori, S Kyo, M Nakamura, M Hashimoto, Y Maida, Y Mizumoto, M Takakura, S Ohno, T Kiyono, M Inoue

**Affiliations:** 1 Department of Obstetrics and Gynecology, Kanazawa University Graduate School of Medical Science, 13-1 Takaramachi, Kanazawa, Ishikawa 920-8641, Japan; 2 IREIIMS, Tokyo Women's Medical University, Tokyo, Japan; 3 Virology Division, National Cancer Research Institute, Tokyo, Japan

**Keywords:** HER-2, endometrial cancer, paclitaxel, survival

## Abstract

**Background:**

Disabled phosphatidylinositol 3-kinase (PI3K)/AKT and mitogen-activated protein kinase/extracellular signal-regulated kinase signalling is involved in endometrial carcinogenesis, and there is evidence that expression of epidermal growth factor receptor (EGFR) family members has a role in such intracellular signalling pathways. This study analysed the prognostic impact of EGFR family expression in endometrial cancer in relation to PI3K–AKT and MAPK–ERK signalling, as well as drug sensitivity.

**Methods and results:**

Immunohistochemical analysis using 63 surgical specimens of endometrioid-type endometrial cancers revealed that EGFR, human epidermal growth factor receptor (HER)-2 and HER-4 were expressed in 25 (39.7%) of 63, 26 (41.3%) of 63 and 31 (49.2%) of 63 tumours, respectively. Gene amplification of *HER-2* was observed in 2 of 26 patients with high HER-2 expression. Kaplan–Meier analysis revealed that high HER-2 expression was a factor that negatively influenced the progression-free and overall survival rate (*P*<0.05), and multivariate analysis showed high HER-2 expression to be an independent prognostic factor. Subsequently, we performed *in vitro* knockdown analysis to investigate the linkage between HER-2 expression and PI3K–AKT pathways. Short interfering RNA (siRNA)-based knockdown of *HER-2* in endometrial cancer cells led to a significant reduction in phosphorylated AKT (p-AKT) expression, indicating the existence of a HER-2/PI3K-AKT axis. As the PI3K–AKT pathway is known to have crucial roles in anticancer drug sensitivity, we examined the involvement of HER-2 in sensitivity to paclitaxel. Short interfering RNA-based knockdown of *HER-2* conferred increased sensitivity to paclitaxel in endometrial cancer cells, attenuating the induction of p-AKT on paclitaxel stimulation, which was cancelled by inactivating AKT by the introduction of a dominant-negative form.

**Conclusion:**

HER-2 is a significant prognostic factor of endometrioid-type endometrial cancer, as well as a key molecule that affects paclitaxel sensitivity by HER-2 interaction with the PI3K–AKT pathway.

Endometrial cancer is the second most common gynaecological malignancy in Japan, and its incidence in Japan has increased dramatically over the last decade. Although premalignant lesions of endometrial cancer have been well characterised, the molecular pathways of endometrial carcinogenesis remain unclear. Previous studies have identified various genetic mutations in endometrial cancer, including *PTEN*, *PIK3CA* and *KRAS*. The *PTEN* mutation is the most common genetic abnormality detected in endometrioid-type endometrial cancer (Tashiro *et al*, 1997; Mutter *et al*, 2000; Kanaya *et al*, 2005). The fundamental *in vivo* role of PTEN is to inhibit the phosphatidylinositol 3-kinase (PI3K)–AKT pathway. Mutation of *PTEN* can disable this inhibitory function, inducing the antiapoptotic pathway. Recently, mutation of a novel oncogene, *PIK3CA*, was discovered in multiple human epithelial cancers, including endometrial carcinoma (Oda *et al*, 2005; Hayes *et al*, 2006; Velasco *et al*, 2006); this oncogene encodes the catalytic p110*α* subunit of PI3K in various cancers (Samuels *et al*, 2004). The mutant proteins have been shown to display enhanced lipid-kinase activity. These findings show the critical roles that the PI3K–AKT pathway has in endometrial carcinogenesis. Another prevalent genetic abnormality in endometrial carcinogenesis is mutation of the *KRAS* gene (Mizuuchi *et al*, 1992; Enomoto *et al*, 1993). The extracellular signal-regulated kinase–mitogen-activated protein kinase (ERK–MAPK) pathway is activated by mitogenic stimuli mediated by receptor-type tyrosine kinases and G-protein-coupled receptors, leading to sequential phosphorylation of RAS, RAF, MEK and ERK1/2. Phosphorylated ERK translocates to the nucleus and regulates a range of substrates that promote cell proliferation, motility, differentiation and survival (Marshall, 1999; Chang and Karin, 2001; Shaw and Cantley, 2006). A *KRAS* mutation can lead to continuous stimulation of its downstream targets, resulting in ERK1/2 activation in the absence of mitogenic stimuli. Thus, most of these prevalent genetic alterations observed in endometrial cancer stimulate the PI3K–AKT and MAPK–ERK pathways, which may have major roles in endometrial carcinogenesis. However, the upstream pathways for their activation remain unclear.

The EGFR family consists of four members, EGFR (human epidermal growth factor receptor (HER)-1), HER-2, HER-3 and HER-4, which share structural homology consisting of an extracellular domain and a cytoplasmic signal-transduction domain with tyrosine kinase activity (Olayioye *et al*, 2000). Many kinds of tumour cells express multiple EGFR members, which interact to form an array of homodimers and heterodimers. Human epidermal growth factor receptor-2 is predominantly activated by forming a heterodimer with other EGFR members, rather than forming a homodimer (Goldman *et al*, 1990; Wada *et al*, 1990; Gamett *et al*, 1995). Heterodimerisation of HER-2 can result in the activation of intracellular signalling cascades, such as the PI3K–AKT and MAPK–ERK pathways (Dougall *et al*, 1994; Olayioye *et al*, 2000; Olayioye, 2001; Asanuma *et al*, 2005). However, the roles of the EGFR family in endometrial cancer have not been well investigated, particularly in relation to the PI3K–AKT and MAPK–ERK pathways.

In this study, we examined the status of expression of EGFR family members and their involvement in the PI3K–AKT and MAPK–ERK pathways in endometrial cancer. Furthermore, the role of the EGFR family in patient survival and paclitaxel sensitivity was investigated.

## Materials and methods

### Patients and tissue samples

A total of 63 patients with endometrioid-type endometrial cancer (mean age, 57.5 years; range, 32–78 years) treated at the Department of Obstetrics and Gynecology, Kanazawa University Hospital, from January 1995 to December 2002, were enrolled in the study. All patients underwent a total abdominal or radical hysterectomy plus bilateral salpingo-oophorectomy. Systemic retroperitoneal lymphadenectomy was performed in approximately 70% of patients. Staging was performed for all patients using the International Federation of Gynaecology and Obstetrics (FIGO) surgical staging system: 46 tumours were classified as stage I (substages: Ia, 10 tumours; Ib, 28 tumours; and Ic, eight tumours); six tumours were classified as stage II (substages: IIa, three tumours and IIb, three tumours); nine tumours were classified as stage III (substages: IIIa, three tumours and IIIc, six tumours); and two tumours were classified as stage IV. On the basis of histological examination, 34 tumours were classified as grade (G)1, 14 tumours were classified as G2 and 15 tumours were classified as G3. Patients with deep myometrial invasion, cervical involvement and special histology underwent external radiotherapy, whereas those with positive peritoneal cytology or retroperitoneal lymph-node metastasis were treated with 4–6 cycles of CAP chemotherapy (90 mg m^−2^ cisplatin, 50 mg m^−2^ doxorubicin and 500 mg m^−2^ cyclophosphamide) or TC chemotherapy (180 mg m^−2^ paclitaxel, and carboplatin, according to Chatelut's formula (area under the curve=5 mg ml^−1^ min^−1^)) as postoperative adjuvant therapy. Patient treatment was followed up with a gynaecological examination, recording of laboratory data, transvaginal and abdominopelvic ultrasonography and a radiological examination. Data from regular follow-up visits to the outpatient department were stored in a database designed specifically for endometrial carcinoma patients. A telephone survey to update the status of all surviving patients was carried out in August 2006. The exact date of disease recurrence was defined as the date when apparent tumours were detected by ultrasonographic or radiological examinations. Tumour samples were collected at the time of surgery, with written informed consent from patients and approval of the Ethics Committee of Kanazawa University. Half of each tissue sample was examined histologically by pathologists, and the remaining portion of each sample was frozen at −80°C until DNA extraction for mutation analysis.

### Mutation analysis

Of the 63 endometrial cancers, 45 were available for DNA sequencing and had been evaluated previously for mutations in *PTEN*, *PIK3CA* and *KRAS (*Kanaya *et al*, 2005; Mizumoto *et al*, 2007; Mori *et al*, 2007). All exons of the *PTEN* gene, exons 9 and 20 of the *PIK3CA* gene and exon 1 of the *KRAS* gene (including codons 12 and 13) were amplified by polymerase chain reaction (PCR) using primer sets described previously (Kanaya *et al*, 2005; Mizumoto *et al*, 2007; Mori *et al*, 2007). Polymerase chain reaction products were purified using the Qiagen PCR purification kit (Qiagen, Valencia, CA, USA), and direct sequencing was performed. Polymerase chain reaction products with suspected mutations were reamplified and subsequently cloned into the pGEM-T Easy vector (Promega, Mannheim, Germany), and sequencing was performed using at least three clones.

### Immunohistochemistry

Immunohistochemical analysis was performed using formalin-fixed, paraffin-embedded specimens from the 63 endometrioid-type endometrial cancer samples with a rabbit polyclonal antibody to HER-2 (code number A0485) (DakoCytomation, Carpinteria, CA, USA), a mouse monoclonal antibody to EGFR (clone EGFR.113) (Novocastra, Newcastle upon Tyne, UK) and a rabbit monoclonal antibody to HER-4 (No. 4792; Cell Signaling Technology, Beverly, MA, USA). Of these, 39 cancers had been evaluated previously using mouse monoclonal antibody to MLH1 (clone G168-15, code 13271A) (PharMingen, San Diego, CA, USA) (Kanaya *et al*, 2005). After the specimens were deparaffinised in xylene and a graded alcohol series, epitope retrieval was performed. The sections were heated in a microwave oven at 700 W for 10 min in 1 × antigen retrieval solution (Biogenex, San Ramon, CA, USA). Endogenous peroxidase was then blocked by immersing the sections in 0.3% H_2_O_2_–methanol for 30 min. After blocking with horse serum, the primary antibodies were diluted at 1 : 20 (for EGFR) or at 1 : 400 (for HER-2 and HER-4) and applied for 16 h at 4°C. The reaction was visualised using the EnVision Detection Kit (Dako Cytomation) using diaminobenzidine tetrahydrochloride as the enzyme substrate. All sections were counterstained with GM haematoxylin stain solution (Muto Pure Chemicals, Tokyo, Japan). For negative controls, isotype control immunoglobulin G (IgG) was used. The staining results for HER-2 expression were scored in accordance with the Hercep Test guidelines (Dako Cytomation): score 0, negative, no staining or staining, but without a membranous pattern; score 1, negative, incomplete membranous staining or complete membranous staining in less than 10% of tumour cells; score 2, positive, complete membranous staining in >10% of tumour cells of moderate intensity; and score 3, positive, complete membranous staining in >10% of tumour cells of strong intensity. The staining results for EGFR and HER-4 expression, in which positive staining is defined as any staining of tumour cell membranes above background level, whether it is complete or incomplete circumferential staining, have been classified into three levels (+, ++, and +++) depending on the staining intensity. Staining for MLH1 was previously classified into three levels: − (positive in <25% of tumour cells), + (positive in 25–75% of tumour cells) or as ++ (positive in >75% of tumour cells) (Kanaya *et al*, 2005). For analysis of the relationship with clinicopathological characteristics, the expression of HER-2 was classified into two levels, namely, low (score 0 or 1) and high (score 2 or 3), whereas that of EGFR/HER-4 or MLH1 was also classified into two levels, namely, low (− or +) and high (++ or +++) or low (− or +) and high (++), respectively. Stained sections were evaluated by two observers with no previous knowledge of the clinicopathological parameters. The average staining score was registered, and there was no statistically significant difference in scoring between observers.

### Chromogenic *in situ* hybridisation

Chromogenic *in situ* hybridisation (CISH) for *HER-2* amplification was carried out in patients with high HER-2 expression using a ZytoDot SPEC HER-2 Probe kit (ZytoVision, Bremerhaven, Germany) in accordance with the manufacturer's guidelines, and performed manually. The sections were evaluated with the Olympus BX51 microscope (Olympus Optical Company Ltd., Tokyo, Japan) using a × 40 dry objective. A nonamplified gene copy number was defined as 1–5 signals per nucleus. Amplification was defined as six or more signals per nucleus in more than 50% of cancer cells, or when a large gene copy cluster was seen.

### Cell lines and cultures

Human endometrial cancer cells (HEC1A and Ishikawa) were obtained from ATCC (Manassas, VA, USA). Human endometrial epithelial immortalised cells (EM-E6/E7/TERT) were previously established by us (Kyo *et al*, 2003). EM-E6/E7/TERT cells with inactive AKT were established as follows: First, the mutant *AKT* cDNA expressing the dominant-negative form of AKT was excised from vector pUSEamp-Akt1(K179) (Millipore, Billerrica, MA, USA) and subcloned into the retroviral vector pCMSCVpuro, which was then stably transfected into EM-E6/E7/TERT cells, generating EM-E6/E7/TERT/DN-AKT cells. Cells were maintained in Dulbecco's modified Eagle's medium supplemented with 10% fetal bovine serum and 1% penicillin/streptomycin in an atmosphere of 5% CO_2_ at 37°C.

### Knockdown study of *human epidermal growth factor receptor-2*

Cells were seeded and transfected with 30 nM of negative control short interfering RNA (siRNA) or human HER-2 siRNA oligonucleotides (Applied Biosystems, Foster City, CA, USA) using HiPerFect Transfection Reagent (Qiagen) according to the manufacturer's protocol.

### Chemosensitivity assay

A total of 4 × 10^3^ cells (Ishikawa and EM-E6/E7/TERT) or 8 × 10^3^ cells (HEC1A) were seeded in 96-well plates, incubated with siRNA to target *HER-2* for 48 h and then treated with 10 nM paclitaxel (Bristol Pharmaceuticals, Bristol, UK) for different time periods. After each incubation, 1 *μ*g of WST-1 reagent (Roche, Mannheim, Germany) was added to each well, and the cells were further incubated for 2 h at 37°C. Absorbance at wavelengths between 420 and 480 nm was then measured with a microplate reader and WST-1 activity was determined to evaluate chemosensitivity. The experiments were conducted in triplicate.

### Western blot analysis

Whole-cell extracts were prepared as described previously (Mizumoto *et al*, 2007; Mori *et al*, 2007) and 50 *μ*g of extracts was then electrophoresed through a sodium dodecylsulphate–polyacrylamide gel and transferred to a polyvinylidene difluoride membrane. Membranes were blocked with TBS-T (150 mM NaCl, 20 mM Tris-HCl (pH 7.5) and 0.1% Tween 20) containing 5% nonfat dried milk, and were then incubated with a specific primary antibody against HER-2 (No. 2242; Cell Signaling Technology), or with phosphorylated AKT (p-AKT) (No. 4058; Cell Signaling Technology), AKT (No. 9272; Cell Signaling Technology) or FOXO3a (No. 9467; Cell Signaling Technology), followed by incubation with horseradish peroxidase-linked anti-rabbit IgG. Immunoreactive bands were visualised using the ECL detection system (GE Amersham Bioscience, Freiburg, Germany), according to the manufacturer's instructions. As an internal control for equal protein loading, *β*-actin expression was examined simultaneously using a specific antibody (sc-1615; Santa Cruz Biotechnology, Santa Cruz, CA, USA). Relative amounts of each protein were quantified using NIH Image software.

### Statistical analysis

Statistical analysis was performed using the statistical package StatView version 5.0 (Abacus Concepts, Berkeley, CA, USA). In the *in vivo* study, we used the *χ*^2^-test for 2 × 2 tables to compare the categorical data. Survival curves were computed using the Kaplan–Meier method, whereas the log-rank test was used to assess statistical significance. Cox's proportional hazards regression model in a stepwise manner was used to analyse the independent prognostic factors. For *in vitro* results, all values represent mean±s.d. Statistical significance between two groups was determined using a two-tailed *t*-test. A *P*-value <0.05 was considered to indicate statistical significance.

## Results

### Expression of epidermal growth factor receptor family members and correlation with clinicopathological and genetic characteristics in endometrial cancer

**Figure 1:**
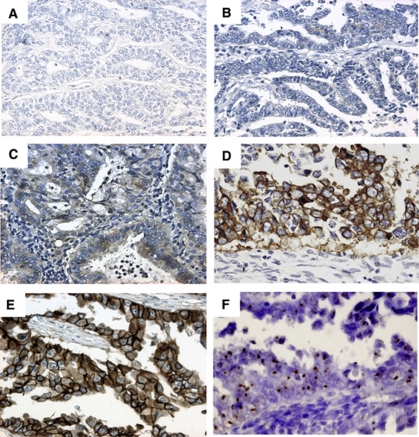
Immunohistochemistry of HER-2 in endometrioid-type endometrial cancer. Representative results of each score are shown (**A**: score 0; **B**: score 1; **C**: score 2 ( × 200) and **D**: score 3 ( × 400)). The definition of each score is described in Materials and Methods. A breast cancer specimen (**E**) with HER-2 expression was used as the positive control for appropriate staining conditions ( × 400). (**F**) CISH analysis of the case (**D**) showing high levels of *HER-2* amplification ( × 400). Brown dots show the large gene copy clusters.

We first investigated the expression of EGFR family members EGFR, HER-2 and HER-4 by immunohistochemistry using 63 surgical specimens of endometrioid-type endometrial cancers, on the basis of the known finding that HER-3 expression is not upregulated in endometrial cancer (Ejskjaer *et al*, 2007). The expression of HER-2 was classified into two levels, namely, low (score 0 or 1) and high (score 2 or 3), whereas that of EGFR/HER-4 was also classified into two levels, namely, low (− or +) and high (++ or +++). High expressions of EGFR, HER-2 and HER-4 were observed in 25 (39.7%) of 63, 26 (41.3%) of 63 and 31 (49.2%) of 63 tumours, in which 48.0% (12 out of 25), 61.5% (16 out of 26) and 54.8% (17 out of 31), respectively, exhibited heterogeneous expression within tumours. There was no preferential expression of these members in any of the specific tumour regions, such as in the invasive front or at the centre of tumours or in areas of squamous differentiation. Representative staining patterns of HER-2 are shown in Figure 1. *Human epidermal growth factor receptor-2* gene amplification was further analysed in 26 patients with a high HER-2 expression by CISH, and two patients (FIGO1b, G1 and FIGO3c, G2, respectively) with score 3 HER-2 expression were found to have *HER-2* gene amplification. We next examined the relationship between these expression patterns and the clinicopathological characteristics of the specimens (Table 1). However, no statistically significant correlation was observed between them.

We then examined the correlation of these expression patterns and the mutation status of *PTEN*, *PIK3CA* and *KRAS*, on the basis of our previous data of the frequency of such mutations in an overlapped population of endometrial cancers (Table 2). Among the 45 patients from whom DNA samples were available, 18 (40%) had a *PTEN* mutation, 7 (15.6%) had a *PIK3CA* mutation and 10 (22.2%) had a *KRAS* mutation. No correlation was observed between the positivity of these factors and the mutation of each gene. However, a high HER-2 expression was likely to have wild-type *PTEN* (*P*=0.079). Notably, strong HER-2 expression with a score of 3 was significantly associated with wild-type *PTEN* (*P*=0.032) (Table 3).

We further examined the correlation of expression of EGFR family members with MLH1, a representative mismatch repair protein, loss of expression of which correlates with mutation of *PTEN* or other genes involved in endometrial carcinogenesis. No significant association was observed between them, but there was a tendency for high HER-2 expression to be associated with high MLH1 expression, although the difference did not reach statistical significance (*P*=0.0746) (Table 4).

### Survival impact of epidermal growth factor receptor family expression in endometrial cancer

**Figure 2:**
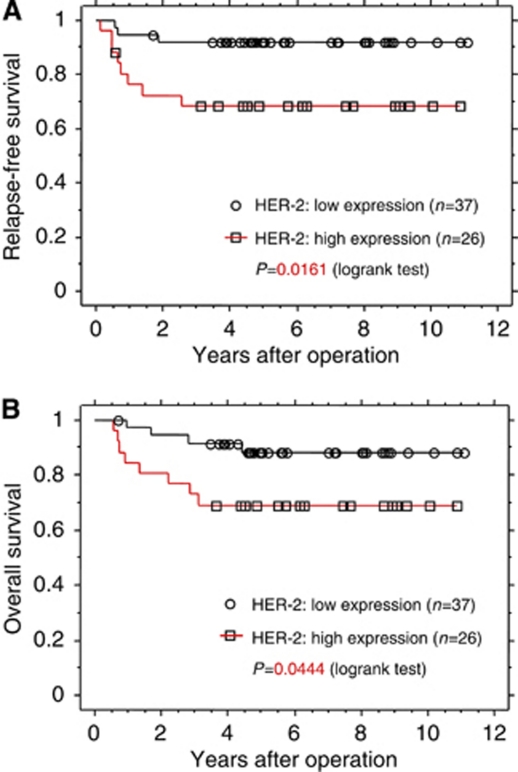
Kaplan–Meier survival curves of 63 patients with endometrial cancer in relation to HER-2 expression. (**A**) Relapse-free survival (**B**) Overall survival.

Next, the survival impact of EGFR family member expression was examined by Kaplan–Meier analysis (Figure 2). The median follow-up for all patients was 5.16 years (range, 0.58–11.08 years). Among the 63 patients, 11 patients (17.5%) had relapses of endometrial cancer at the time of last follow-up and 12 patients (19.0%) died. When HER-2 staining scores were used as cutoff points to stratify patients into two groups (see Materials and Methods), the progression-free survival (PFS) for low (score 0 or 1) and high (score 2 or 3) HER-2 expression was 91.9 and 69.2%, respectively (*P*=0.016). The overall survival (OS) for low and high HER-2 expression was 89.2 and 69.2%, respectively (*P*=0.044). Thus, high HER-2 expression was a factor that negatively influenced PFS and OS rates by univariate analysis. Epidermal growth factor receptor and HER-4 expression levels were not factors that affected PFS or OS by univariate analysis (Supplementary Figure 1). When other known variables for prognosis of endometrial cancer, including FIGO stage, pathological grade and myometrial invasion, were included in a Cox proportional hazard analysis for relapse-free survival, HER-2 expression (hazard ratio 5.31, *P*=0.0180) and FIGO stage were identified as independent predictive factors of patient survival (Table 5).

### The phosphatidylinositol 3-kinase–AKT pathway may be a downstream target of human epidermal growth factor receptor-2 in endometrial cancer

**Figure 3:**
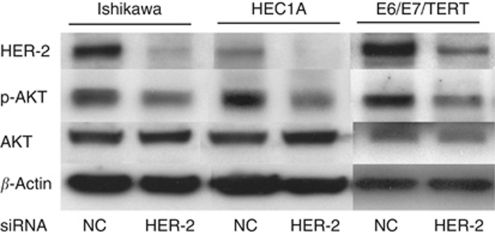
Linkage between HER-2 and p-AKT expressions in endometrial cancer or immortalised cells. Endometrial cancer Ishikawa and HEC1A cells or endometrial immortalised EM-E6/E7/TERT cells were treated with or without siRNA against *HER-2*. After 48 h, cell lysates were prepared and western blot analyses were performed with antibodies against HER-2, p-AKT and total AKT. NC: negative control sample treated with scrambled siRNA.

The above findings indicate that HER-2 expression is a critical prognostic factor in endometrial cancer. We next sought to identify the downstream target of HER-2 expression using various endometrial cancer cell lines or immortalised endometrial epithelial cells (EM-E6/E7/TERT). One possible candidate for a downstream target of HER-2 was the PI3K–AKT pathway, based on previous analyses in other tumour types (Knuefermann *et al*, 2003; Qi *et al*, 2009); hence, we focused on this pathway. To examine the linkage between HER-2 and the PI3K–AKT pathway, a knockdown of *HER-2* was performed in the endometrial cancer cell line Ishikawa, using siRNA techniques, and the expression of p-AKT was evaluated by western blot analysis. As shown in Figure 3, knockdown of *HER-2* was confirmed to be efficient, with a more than 50% reduction in expression. Notably, p-AKT expression concomitantly decreased with *HER-2* knockdown. This finding was not limited to Ishikawa cells, as both endometrial cancer HEC1A cells and EM-E6/E7/TERT cells exhibited a similar decrease in p-AKT expression on *HER-2* knockdown (Figure 3). Thus, the HER-2 pathway clearly links to the PI3K–AKT pathway in endometrial cancer cells.

We examined the status of mutation in *PTEN*, *PIK3CA* and *KRAS* genes in these cell lines by DNA sequencing, and found a frameshift mutation in exon 8 of the *PTEN* gene (319ACTT del, 289A del) in Ishikawa cells and missense mutations in exon 20 of the *PIK3CA* gene (G3145C), as well as in exon 1 of the *KRAS* gene (G35A) in HEC1A cells as previously reported (Kim *et al*, 2004; Oda *et al*, 2005). We found no mutation of these genes in EM-E6/E7/TERT cells. These findings indicate that the linkage of HER-2 with the PI3K–AKT pathway is maintained irrespective of the mutation status of such genes.

### Human epidermal growth factor receptor-2 expression is involved in paclitaxel sensitivity in endometrial cancer cells by regulation of the phosphatidylinositol 3-kinase –AKT pathway

**Figure 4:**
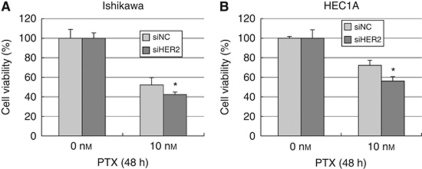
Increased sensitivity to paclitaxel by knockdown of *HER-2*. Endometrial cancer Ishikawa (**A**) and HEC1A cells (**B**) were incubated with siRNA against *HER-2* (siHER-2) or scrambled siRNA (siNC) for 48 h. Cells were then treated with 10 nM of paclitaxel for an additional 48 h, followed by the WST-1 assay to examine cell viability. The viability of untreated cells incubated with siNC was set as the control level (100%) and the percentage of cell viability in each cell type was normalised relative to the untreated control. Each experiment was performed in triplicate in three independent experiments. Columns, mean; bars, ±s.d. ^*^P<0.05.

**Figure 5:**
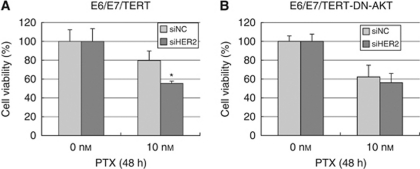
Inactivation of AKT cancels the effect of HER-2 on paclitaxel sensitivity. Endometrial immortalised EM-E6/E7/TERT cells (**A**) or those with the dominant-negative form of AKT (**B**) were incubated with siRNA against *HER-2* (siHER-2) or scrambled siRNA (siNC) for 48 h. Cells were then treated with 10 nM of paclitaxel for an additional 48 h, followed by the WST-1 assay to examine cell viability. The viability of untreated cells incubated with siNC was set as the control level (100%) and the percentage of cell viability in each cell type was normalised relative to the untreated control. Each experiment was performed in triplicate in three independent experiments. Columns, mean; bars, ±s.d. ^*^P<0.05.

There is accumulating evidence that the PI3K–AKT pathway is critically involved in drug resistance to chemotherapies of various types of cancers by activation of survival signals. Linkage of HER-2 to p-AKT expression prompted us to examine the involvement of HER-2 expression in the chemosensitivity of endometrial cancer cells. We therefore sought to test the sensitivity to paclitaxel, a key drug for endometrial cancer cells. First, preparatory experiments were conducted to test the efficacy of paclitaxel against Ishikawa, HEC1A and immortalised endometrial epithelial cells, in which cells were treated with 1–1000 nM of paclitaxel and cell viability was measured by WST-1 assay. We found that 10 or 100 nM was an ideal concentration to elicit specific effects of paclitaxel in each cell type examined (data not shown). On the basis of these findings, all three cell lines were treated with or without HER-2 siRNA and exposed to 10 nM of paclitaxel for 48 h. Cell viability was then examined by WST-1 assay. As shown in Figures 4 and 5A, *HER-2* knockdown significantly augmented the cytotoxic effect of paclitaxel in each cell type.

**Figure 6:**
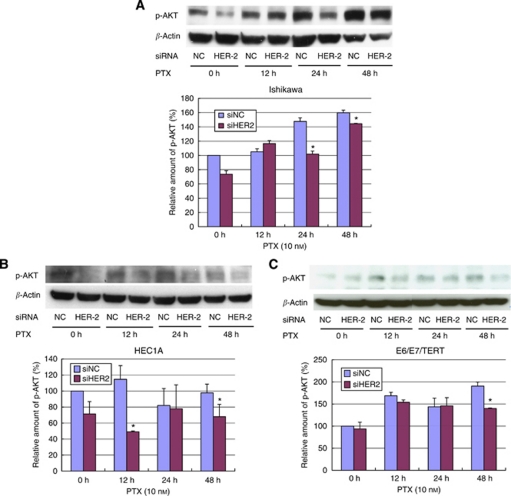
Induction of p-AKT expression on paclitaxel treatment is attenuated by *HER-2* knockdown. Endometrial cancer Ishikawa (**A**) and HEC1A cells (**B**) or immortalised EM-E6/E7/TERT cells (**C**) were incubated with siRNA against *HER-2* (siHER-2) or scrambled siRNA (siNC) for 48 h. Cells were then treated with 10 nM of paclitaxel for an additional 48 h. Cell lysates were prepared and western blot analyses were performed with antibodies against p-AKT. NC: negative control sample treated with scrambled siRNA. A representative image of the bands is shown. Bband intensity was measured using NIH image and is represented as the relative amount of p-AKT after normalisation against *β*-actin expression. Each experiment was performed in triplicate in three independent experiments. Columns, mean; bars, ±s.d. ^*^P<0.05.

Next, we monitored the expression of p-AKT during treatment with paclitaxel in each cell type by western blot analysis. In Ishikawa cells, p-AKT expression increased on stimulation of paclitaxel at 24–48 h after treatment (Figure 6A). We also confirmed the expression of FOXO3a, a known downstream target of p-AKT. FOXO3a expression began to increase at 48 h and this upregulation lasted until at least 96 h (data not shown). Thus, we confirmed the functional activation of AKT during treatment with paclitaxel. We also tested whether HER-2 expression affects the induction of p-AKT. Ishikawa cells were treated with 10 nM paclitaxel under knockdown of *HER-2*, and p-AKT expression was then monitored. Figure 6 shows that cells with *HER-2* knockdown led to decreased levels of p-AKT expression, concomitant with increased sensitivity to paclitaxel, as shown in Figure 4. In HEC1A cells, p-AKT expression increased on stimulation with paclitaxel at 12 h after treatment (Figure 6B), and *HER-2* knockdown led to a significant decrease in p-AKT expression. The levels of p-AKT at 24 or 48 h were unstable, but apparently decreased on *HER-2* knockdown at 48 h as well. In EM-E6/E7/TERT cells, p-AKT expression also increased on stimulation with paclitaxel at 12 h or later after treatment (Figure 6C), and *HER-2* knockdown cancelled this elevation at 48 h. Thus, *HER-2* knockdown inhibited paclitaxel-induced activation of p-AKT expression in endometrial cancer or immortalised cell lines. Interestingly, in HEC1A cells, *HER-2* knockdown at 48 h after treatment, when p-AKT induction was no longer observed, enhanced the sensitivity to paclitaxel (Figure 4B), indicating that the basal levels of p-AKT expression have certain roles in inhibiting the cytotoxic effect of paclitaxel.

We further sought to confirm whether the increased sensitivity to paclitaxel by knockdown of *HER-2* is primarily because of the suppression of p-AKT expression. We prepared another immortalised cell in which EM-E6E7/TERT cells were stably transfected with the dominant-negative form of the *AKT* gene (EM-E6/E7/TERT/DN-AKT cells) (see Materials and Methods), so that they have inactivated AKT function. Using these cells, we performed the paclitaxel sensitivity test by the WST-1 assay. As shown in Figure 5B, introduction of dominant-negative AKT largely cancelled the enhanced sensitivity to paclitaxel achieved by the knockdown of *HER-2*. Thus, the effect of HER-2 seems to be primarily dependent on AKT activity. Taken together, we conclude that HER-2 expression has a critical role in the induction of p-AKT expression, thereby affecting the sensitivity of endometrial cancer cells to paclitaxel.

## Discussion

In this study, we found frequent expressions of EGFR, HER-2 and HER-4 approximately in 40–50% of endometrioid-type endometrial cancer. There was no significant association between expressions of these three factors and the clinicopathological characteristics of endometrial cancer. These findings are in contrast to a previous study by Morrison *et al* (2006), in which HER-2 expression was associated with high-grade cancers (G3 and nonendometrioid types) or with higher stages (FIGO stages IIIa–IVb). This discrepancy may at least partly be accounted for by the difference in sample population between the studies. Our study examined endometrioid-type cancers, whereas Morrison *et al* (2006) investigated a much larger percentage (39%) of type 2 cancers. Nevertheless, we unexpectedly found that EGFR family expression was not closely associated with the clinicopathological characteristics of endometrioid-type endometrial cancer in Japanese patients. The rate of *HER-2* amplification in our study was low, with only 2 of 26 high-expression patients having *HER-2* amplification. This is in accordance with Morrison's report that endometrioid-type cancers have low frequency of *HER-2* amplification (1% in G1, 3% in G2 and 8% in G3 cancers) (Morrison *et al*, 2006). Owing to the small number of samples with *HER-2* amplification, its relationship with clinicopathological characteristics remains unclear.

The association between the EGFR family and other genetic alterations involved in endometrial carcinogenesis was our next point of focus. We tested the association between mutations of three genes representative of common genetic mutations in endometrial cancers: *PETN*, *PIK3CA* and *KRAS*. Among the factors examined, there was a tendency for HER-2 expression to be associated with wild-type *PTEN* (*P*=0.079). In particular, strong HER-2 expression was closely linked to wild-type *PTEN* (*P*=0.032). These findings suggest that HER-2 signalling is distinct from the *PTEN* mutation pathway. In the present siRNA knockdown study, we found that HER-2 expression was closely associated with p-AKT expression, indicating that HER-2 signalling may be upstream of the PI3K–AKT pathway. These findings suggest that HER-2 signalling activates the PI3K–AKT pathway in a PTEN-independent manner. This scenario is easily understood, as the *PTEN* mutation may not be required to activate the PI3K–AKT pathway if an HER-2/PI3K–AKT axis exists. Another interesting finding was the tendency for HER-2 expression to be associated with intact MLH1 expression (*P*=0.0746). We previously showed that low MLH1 expression is mainly caused by its promoter inactivation through DNA hypermethylation, which triggers an accumulation of mutations of several genes, including *PTEN* (Kanaya *et al*, 2005). Thus, the possible association between HER-2 and intact MLH1 may also account for HER-2 signalling being able to directly activate the AKT pathway, thereby not requiring mismatch repair deficiency.

Although the *in vitro* knockdown study of HER-2 showed clear association with p-AKT (Figure 3), HER-2 expression *in vivo* was not significantly associated with the expression of p-AKT on the basis of immunohistochemistry (*P*=0.9411, data not shown). However, this *in vivo* result is not anomalous because, in clinically advanced cancers, there are undoubtedly several factors or signalling pathways that regulate p-AKT expression other than the PI3K–AKT pathway. Therefore, the possible linkage between two factors observed in a knockdown study is not always proven by immunohistochemical comparison with clinical cancer samples.

Among the EFGR family, only HER-2 expression is an independent prognostic factor in endometrial cancer. Morrison *et al* (2006) studied larger numbers of patients with a broad spectrum of histological type, grade and stage of endometrial carcinoma, and showed that in univariate analyses, both HER-2 expression and amplification correlated with disease-specific and progression-free survival. In multivariate analyses, HER-2 expression with amplification correlated with overall survival, but not expression without amplification. Overall survival was significantly shorter in patients who overexpressed and/or showed amplification of HER-2 compared with those who did not (Morrison *et al*, 2006). Our data support the findings of Morrison *et al* (2006) and clearly show that it is HER-2 among the EGFR family that significantly affects patient survival.

Growth factor receptor-mediated signal transduction and PI3K–AKT activation have been implicated in conferring resistance to conventional chemotherapy in breast cancer cells (Yu *et al*, 1996; Knuefermann *et al*, 2003). In uterine cancer cells (Gagnon *et al*, 2004, 2008) and ovarian cancer cells (Cao *et al*, 2008; Weng *et al*, 2009), the AKT pathway is known to have critical roles in chemoresistance. The linkage of HER-2 and p-AKT expression by our siRNA knockdown experiment indicates that HER-2 may be involved in the chemosensitivity of endometrial cancer cells. As expected, HER-2 inhibition by siRNA efficiently showed increased sensitivity to paclitaxel, concomitant with p-AKT inhibition. This increased sensitivity seemed to be at least partly p-AKT dependent, because the increased sensitivity to paclitaxel by *HER-2* knockdown was cancelled in cells with inactive AKT (Figure 5B). Thus, it seems that HER-2 regulates the sensitivity to paclitaxel by modulating AKT activity in endometrial cancer cells. Knuefermann *et al* (2003) reported that, in MCF7 breast cancer cells, overexpression of HER-2 caused a PI3K-dependent activation of AKT, and was associated with an increased resistance of cells to multiple chemotherapeutic agents. They also found that selective inhibition of PI3K or AKT activity sensitised MCF7 breast cancer cells to the induction of apoptosis by chemotherapeutic agents. Our results are consistent with their study and show that HER-2 is one of the key regulators of paclitaxel sensitivity, as an upstream factor of AKT, in endometrial cancer.

In summary, we have shown that, among the EGFR family members, only HER-2 is an independent prognostic factor of endometrioid-type endometrial cancer, which may link to the PI3K–AKT pathway, independent of *PTEN* mutation or mismatch repair deficiency. Our *in vitro* study underscores the importance of the HER-2/PI3K–AKT signalling pathway for regulating the efficacy of paclitaxel in endometrial cancer cells. Therefore, we propose the potential clinical benefit of an appropriate combination of conventional chemotherapeutic drugs with a new generation of signal-transduction inhibitors that target the HER-2/PI3K–AKT pathway for the treatment of endometrial cancer.

## Figures and Tables

**Table 1:** Expressions of EGFR family and clinicopathological characteristics of the patients with endometrial cancer

	**EGFR**			**HER-2**			**HER-4**		
**Variables**	**High**	**Low**	***P*-value**	**High**	**Low**	***P*-value**	**High**	**Low**	***P*-value**
*Age (years)*									
<45 (*n*=7)	2	5	0.5239	1	6	0.1240	3	4	0.7215
⩾45 (*n*=56)	23	33		25	31		28	28	
									
*FIGO stage*									
I (*n*=46)	18	28	0.8829	18	28	0.5705	24	22	0.4383
II–IV (*n*=17)	7	10		8	9		7	10	
									
*Lymph-node metastasis*									
Negative (*n*=58)	24	34	0.3485	25	33	0.3140	28	30	0.6149
Positive (*n*=5)	1	4		1	4		3	2	
									
*Depth (myometrial invasion)*									
a, b (*n*=47)	16	31	0.1168	17	30	0.1588	22	25	0.5141
c (*n*=16)	9	7		9	7		9	7	
									
*Histopathological degree of differentiation*									
Grades 1 and 2 (*n*=48)	17	31	0.2157	20	28	0.9089	22	26	0.3381
Grade 3 (*n*=15)	8	7		6	9		9	6	
									
*Menopause*									
Peri, pre (*n*=23)	6	17	0.0944	6	17	0.0634	10	13	0.4904
Post (*n*=40)	19	21		20	20		21	19	
									
*Body mass index*									
<25 (*n*=38)	17	21	0.3120	18	20	0.2254	22	16	0.0890
⩾25 (*n*=25)	8	17		8	17		9	16	

Abbreviations: EGFR=epidermal growth factor receptor; FIGO=International Federation of Gynaecology and Obstetrics.

**Table 2:** Expressions of EGFR family and genetic mutations of the patients with endometrial cancer

	***PTEN* (*n*=45)**		***PIK3CA* (*n*=45)**		***KRAS* (*n*=45)**	
	**MT (*n*=18)**	**WT (*n*=27)**	**MT (*n*=7)**	**WT (*n*=38)**	**MT (*n*=10)**	**WT (*n*=35)**
*EGFR expression*						
Low (*n*=26)	10	16	6	20	7	19
High (*n*=19)	8	11	1	18	3	16
*P*-value	0.8053		0.1034		0.3749	
						
*HER-2 expression*						
Low (*n*=28)	14	14	5	23	7	21
High (*n*=17)	4	13	2	15	3	14
*P*-value	0.0789		0.5846		0.5651	
						
*HER-4 expression*						
Low (*n*=28)	11	17	6	22	7	21
High (*n*=17)	7	10	1	16	3	14
*P*-value	0.9001		0.1630		0.5651	

Abbreviations: EGFR=epidermal growth factor receptor; HER-2=human epidermal growth factor receptor-2; MT=mutation; WT=wild type.

**Table 3:** Expression of *HER-2* and *PTEN* mutation of the patients with endometrial cancer

	***PTEN* (*n*=45)**	
**HER-2 expression**	**Mutation**	**Wild type**
*Score*		
0	8	11
1	6	3
2	4	7
3	0	6
*P*-value	0.0799	

Abbreviation: HER-2=human epidermal growth factor receptor-2.

**Table 4:** Expressions of EGFR family and MLH1 of the patients with endometrial cancer

	**EGFR (*n*=39)**		**HER-2 (*n*=39)**		**HER-4 (*n*=39)**	
**MLH1 expression**	**Low**	**High**	**Low**	**High**	**Low**	**High**
Low (*n*=30)	16	14	20	10	18	12
High (*n*=9)	7	2	10	6	7	2
Total	23	16	23	16	25	14
*P*-value	0.1910		0.0746		0.3295	

Abbreviations: EGFR=epidermal growth factor receptor; HER-2=human epidermal growth factor receptor-2.

**Table 5:** Cox regression hazard model for relapse-free survival

**Variable**	**Risk factor**	**Univariate (Kaplan-Meier) log-rank test (*P*-value)**	**Multivariate (Cox regression model) hazard ratio (95% confidence interval)**	***P*-value**
FIGO stage	⩾II	<0.0001	6.131 (1.525–24.643)	0.0106
Grade	3	0.0042	3.576 (0.978–13.073)	0.0540
Myometrial invasion	⩾1/2	0.0008	2.138 (0.578–7.909)	0.2548
HER-2 expression	High	0.0161	5.308 (1.332–21.156)	0.0180

Abbreviations: FIGO=International Federation of Gynaecology and Obstetrics; HER-2=human epidermal growth factor receptor-2.
