# Supplementary material for: Expression of HER-2 affects patient survival and paclitaxel sensitivity in endometrial cancer
Source: Br J Cancer. 2010 Jul 27;103(6):889–98. doi: 10.1038/sj.bjc.6605805 (PMC2966616; doi:10.1038/sj.bjc.6605805)
Supplement: Supplementary Figure 1 [file 6605805x1.ppt]

## Slide 1
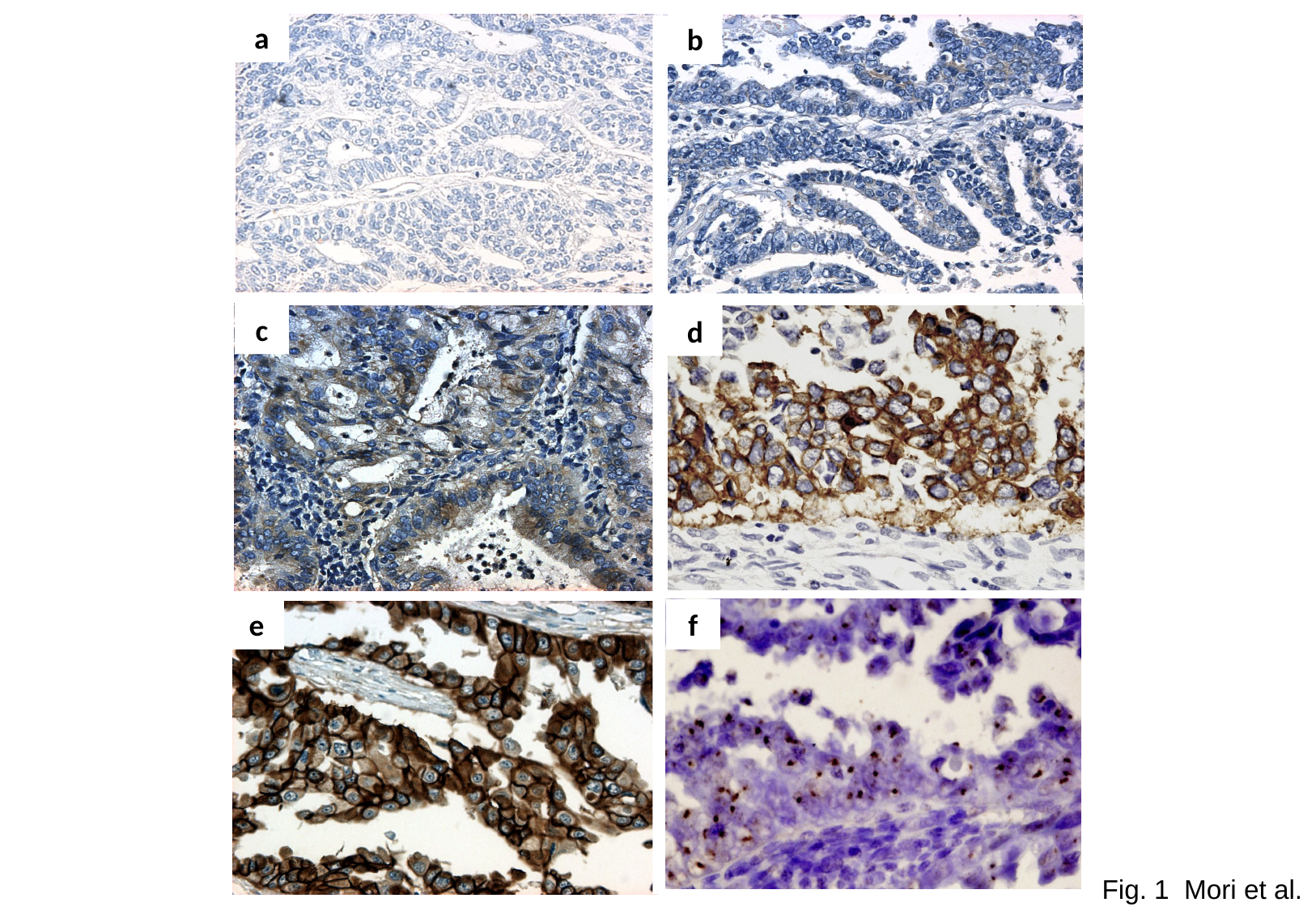

a
b
c
d
e
f
Fig. 1 Mori et al.

## Slide 2
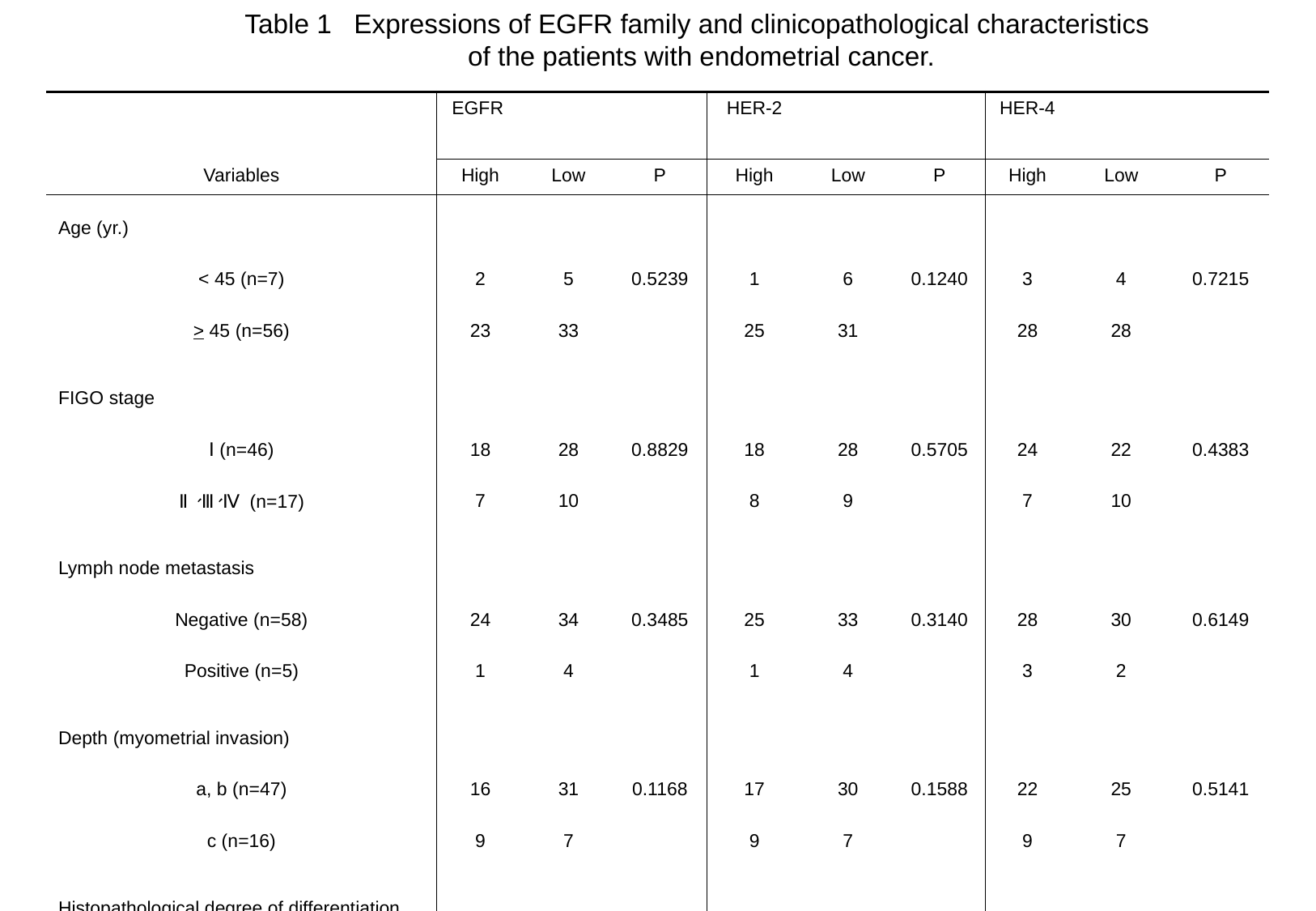

# Table 1 Expressions of EGFR family and clinicopathological characteristics of the patients with endometrial cancer.
| | EGFR | | | HER-2 | | | HER-4 | | |
| --- | --- | --- | --- | --- | --- | --- | --- | --- | --- |
| Variables | High | Low | P | High | Low | P | High | Low | P |
| Age (yr.) | | | | | | | | | |
| < 45 (n=7) | 2 | 5 | 0.5239 | 1 | 6 | 0.1240 | 3 | 4 | 0.7215 |
| > 45 (n=56) | 23 | 33 | | 25 | 31 | | 28 | 28 | |
| FIGO stage | | | | | | | | | |
| Ⅰ (n=46) | 18 | 28 | 0.8829 | 18 | 28 | 0.5705 | 24 | 22 | 0.4383 |
| Ⅱ、Ⅲ、Ⅳ (n=17) | 7 | 10 | | 8 | 9 | | 7 | 10 | |
| Lymph node metastasis | | | | | | | | | |
| Negative (n=58) | 24 | 34 | 0.3485 | 25 | 33 | 0.3140 | 28 | 30 | 0.6149 |
| Positive (n=5) | 1 | 4 | | 1 | 4 | | 3 | 2 | |
| Depth (myometrial invasion) | | | | | | | | | |
| a, b (n=47) | 16 | 31 | 0.1168 | 17 | 30 | 0.1588 | 22 | 25 | 0.5141 |
| c (n=16) | 9 | 7 | | 9 | 7 | | 9 | 7 | |
| Histopathological degree of differentiation | | | | | | | | | |
| Grade 1, 2 (n=48) | 17 | 31 | 0.2157 | 20 | 28 | 0.9089 | 22 | 26 | 0.3381 |
| Grade 3 (n=15) | 8 | 7 | | 6 | 9 | | 9 | 6 | |
| Menopause | | | | | | | | | |
| peri-, pre- (n=23) | 6 | 17 | 0.0944 | 6 | 17 | 0.0634 | 10 | 13 | 0.4904 |
| post (n=40) | 19 | 21 | | 20 | 20 | | 21 | 19 | |
| Body mass index | | | | | | | | | |
| < 25 (n=38) | 17 | 21 | 0.3120 | 18 | 20 | 0.2254 | 22 | 16 | 0.0890 |
| > 25 (n=25) | 8 | 17 | | 8 | 17 | | 9 | 16 | |

## Slide 3
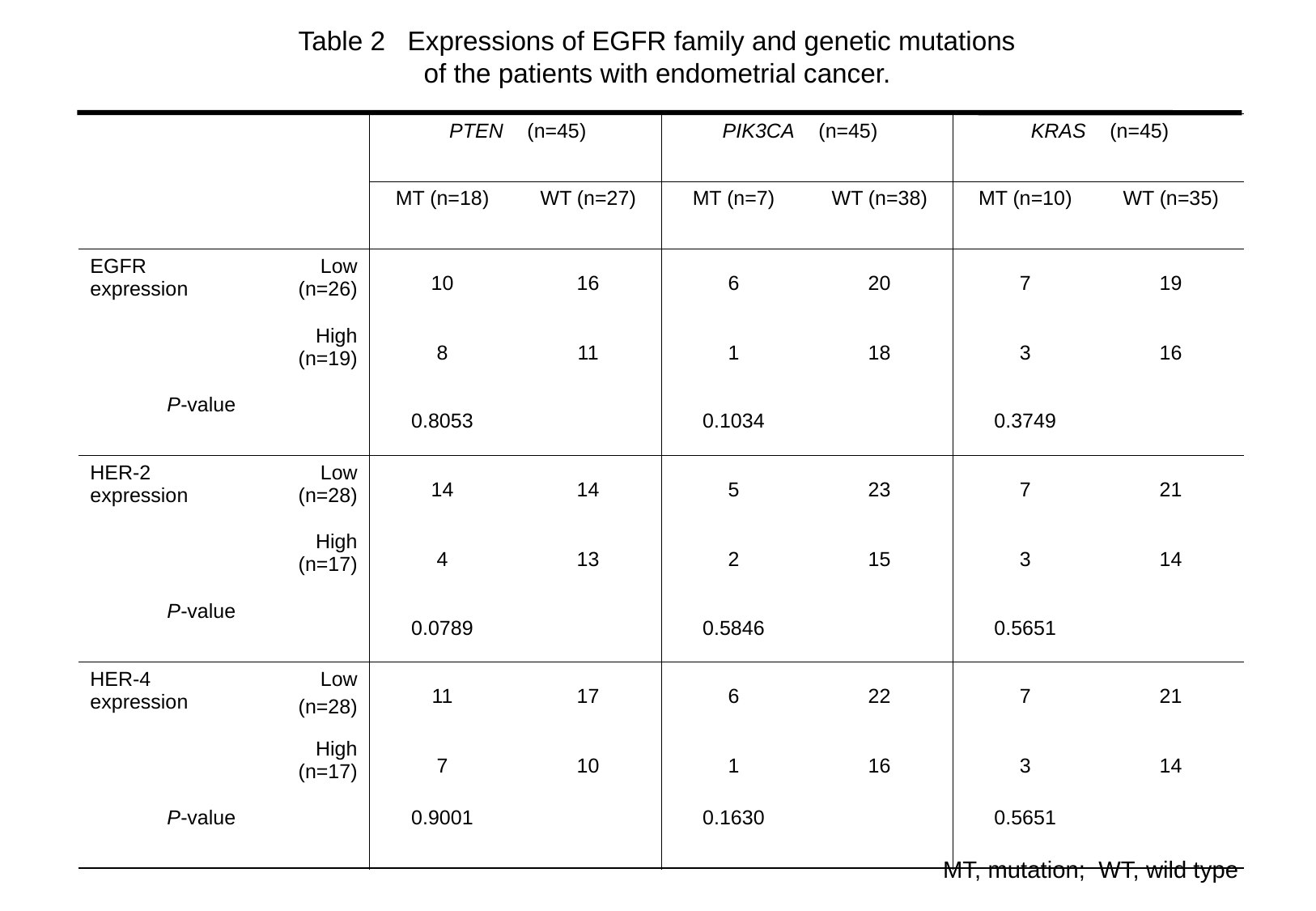

Table 2 Expressions of EGFR family and genetic mutations
of the patients with endometrial cancer.
| | | PTEN | (n=45) | PIK3CA | (n=45) | KRAS | (n=45) |
| --- | --- | --- | --- | --- | --- | --- | --- |
| | | MT (n=18) | WT (n=27) | MT (n=7) | WT (n=38) | MT (n=10) | WT (n=35) |
| EGFR expression | Low (n=26) | 10 | 16 | 6 | 20 | 7 | 19 |
| | High (n=19) | 8 | 11 | 1 | 18 | 3 | 16 |
| P-value | | 0.8053 | | 0.1034 | | 0.3749 | |
| HER-2 expression | Low (n=28) | 14 | 14 | 5 | 23 | 7 | 21 |
| | High (n=17) | 4 | 13 | 2 | 15 | 3 | 14 |
| P-value | | 0.0789 | | 0.5846 | | 0.5651 | |
| HER-4 expression | Low (n=28) | 11 | 17 | 6 | 22 | 7 | 21 |
| | High (n=17) | 7 | 10 | 1 | 16 | 3 | 14 |
| P-value | | 0.9001 | | 0.1630 | | 0.5651 | |
MT, mutation; WT, wild type

## Slide 4
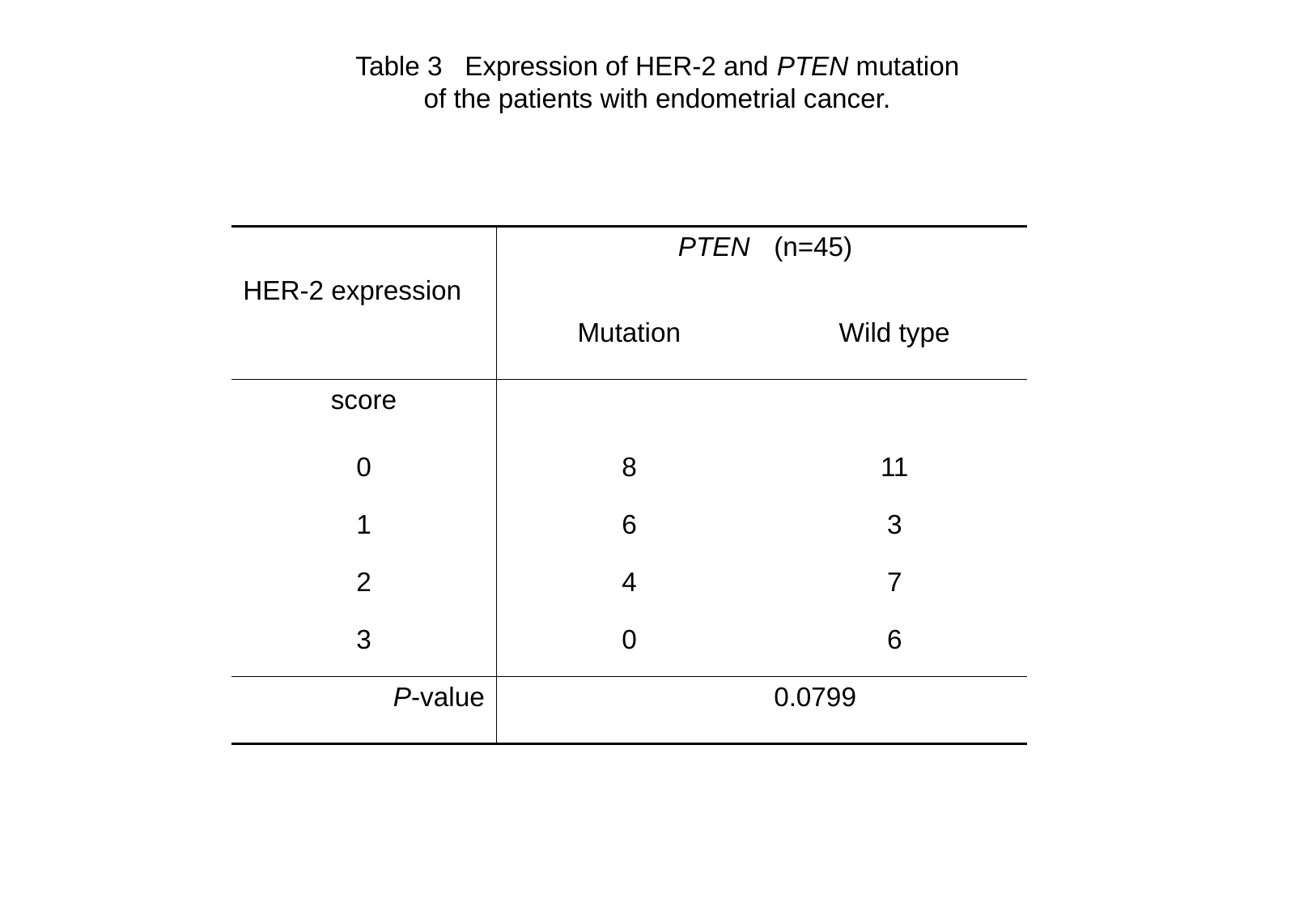

# Table 3 Expression of HER-2 and PTEN mutation of the patients with endometrial cancer.
| HER-2 expression | PTEN | (n=45) |
| --- | --- | --- |
| | Mutation | Wild type |
| score | | |
| 0 | 8 | 11 |
| 1 | 6 | 3 |
| 2 | 4 | 7 |
| 3 | 0 | 6 |
| P-value | | 0.0799 |

## Slide 5
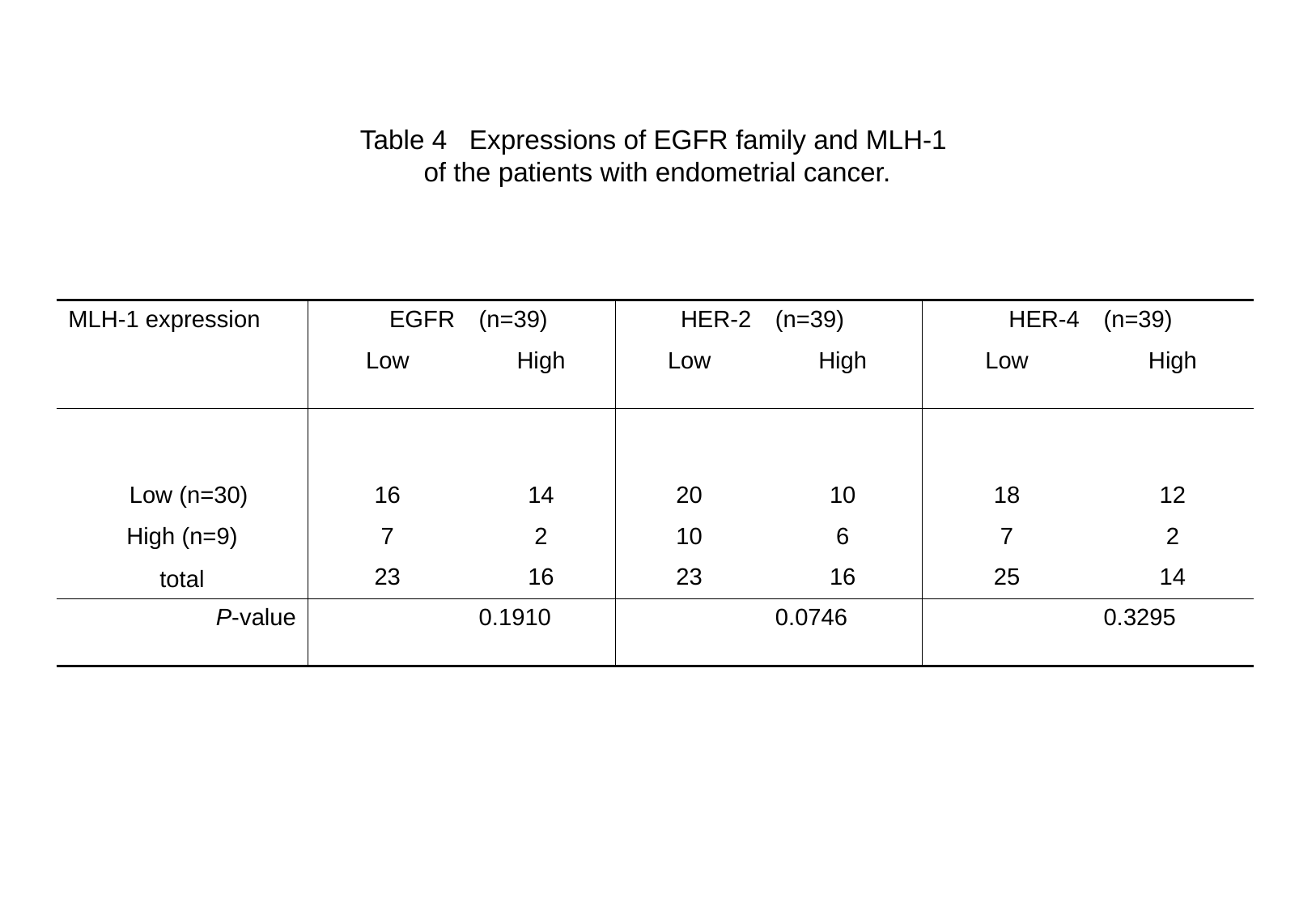

Table 4 Expressions of EGFR family and MLH-1
of the patients with endometrial cancer.
| MLH-1 expression | EGFR | (n=39) | HER-2 | (n=39) | HER-4 | (n=39) |
| --- | --- | --- | --- | --- | --- | --- |
| | Low | High | Low | High | Low | High |
| | | | | | | |
| Low (n=30) | 16 | 14 | 20 | 10 | 18 | 12 |
| High (n=9) | 7 | 2 | 10 | 6 | 7 | 2 |
| total | 23 | 16 | 23 | 16 | 25 | 14 |
| P-value | | 0.1910 | | 0.0746 | | 0.3295 |

## Slide 6
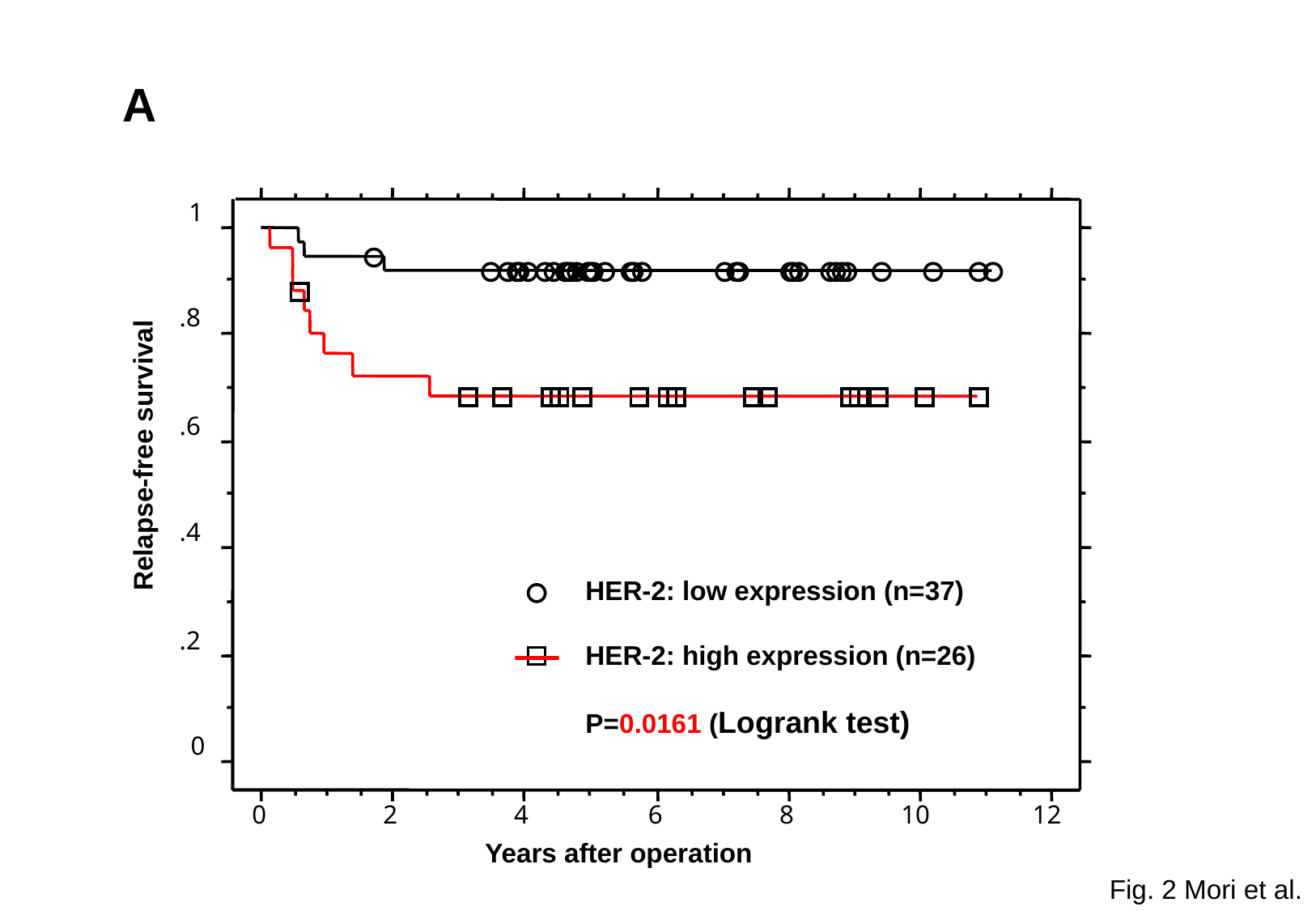

A
1
.8
.6
Relapse-free survival
.4
HER-2: low expression (n=37)
HER-2: high expression (n=26)
P=0.0161 (Logrank test)
.2
0
0
2
4
6
8
10
12
Years after operation
Fig. 2 Mori et al.

## Slide 7
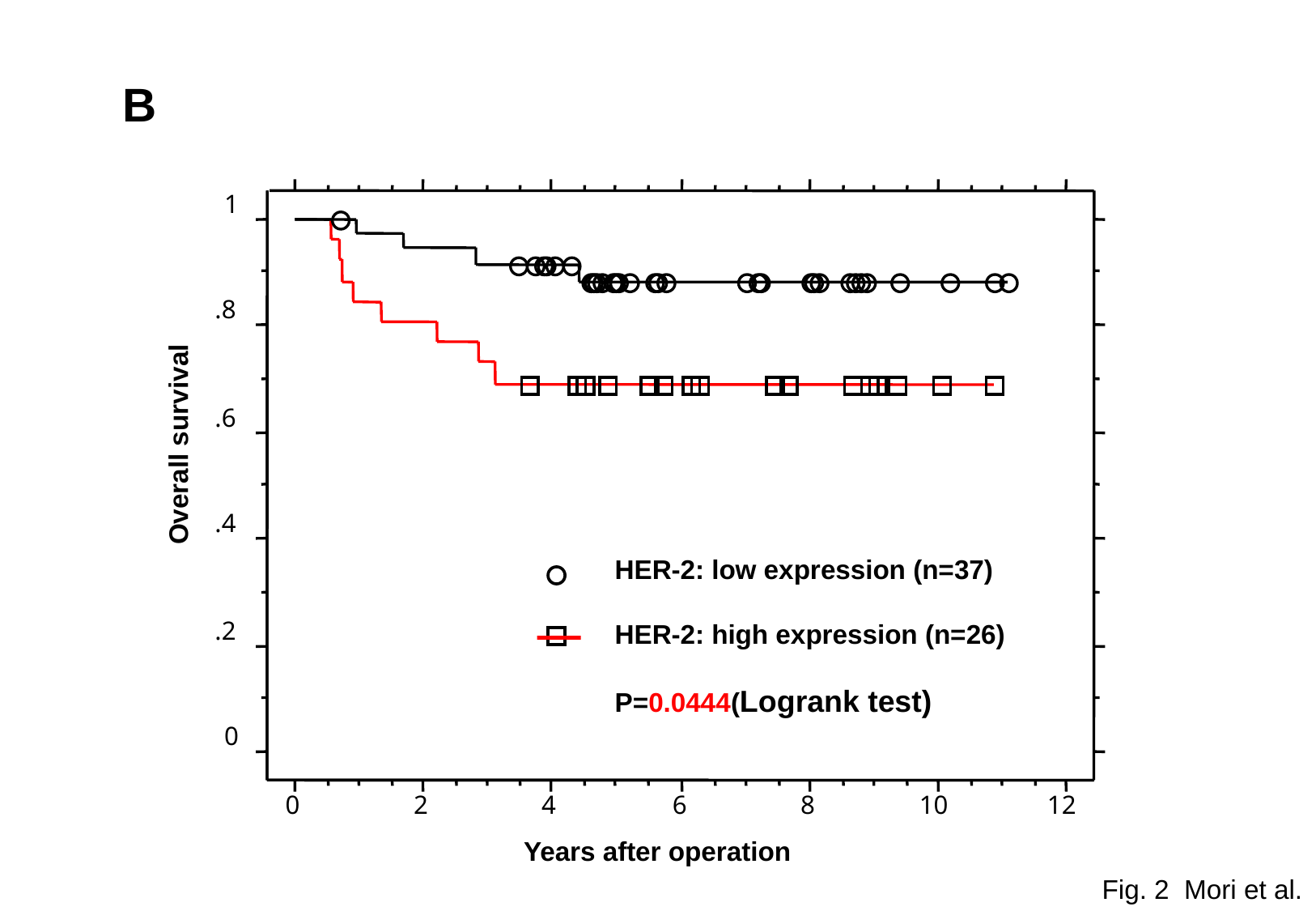

B
1
.8
.6
Overall survival
.4
HER-2: low expression (n=37)
HER-2: high expression (n=26)
P=0.0444(Logrank test)
.2
0
0
2
4
6
8
10
12
Years after operation
Fig. 2 Mori et al.

## Slide 8
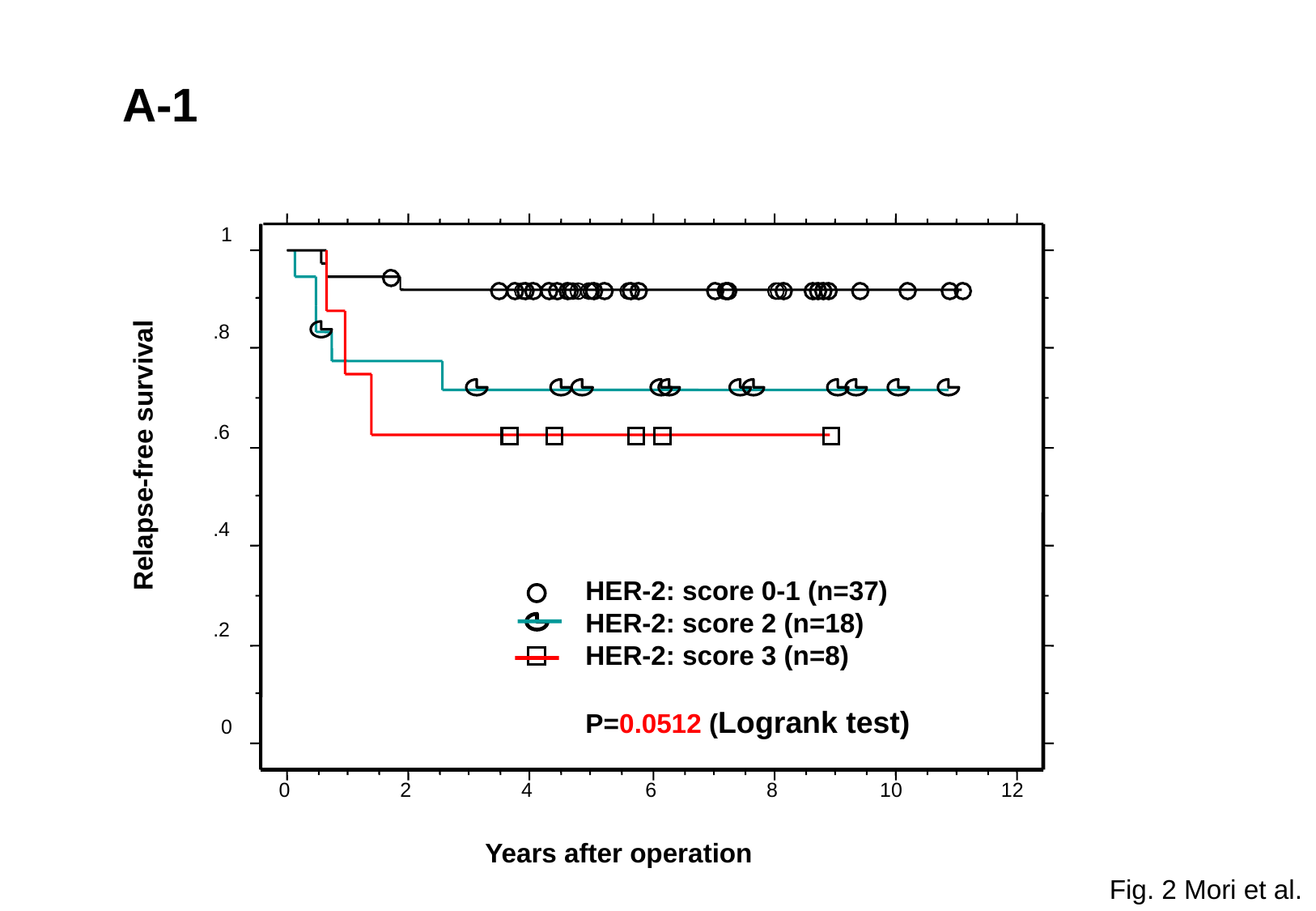

A-1
1
.8
.6
Relapse-free survival
.4
HER-2: score 0-1 (n=37)
HER-2: score 2 (n=18)
HER-2: score 3 (n=8)
P=0.0512 (Logrank test)
.2
0
0
2
4
6
8
10
12
Years after operation
Fig. 2 Mori et al.

## Slide 9
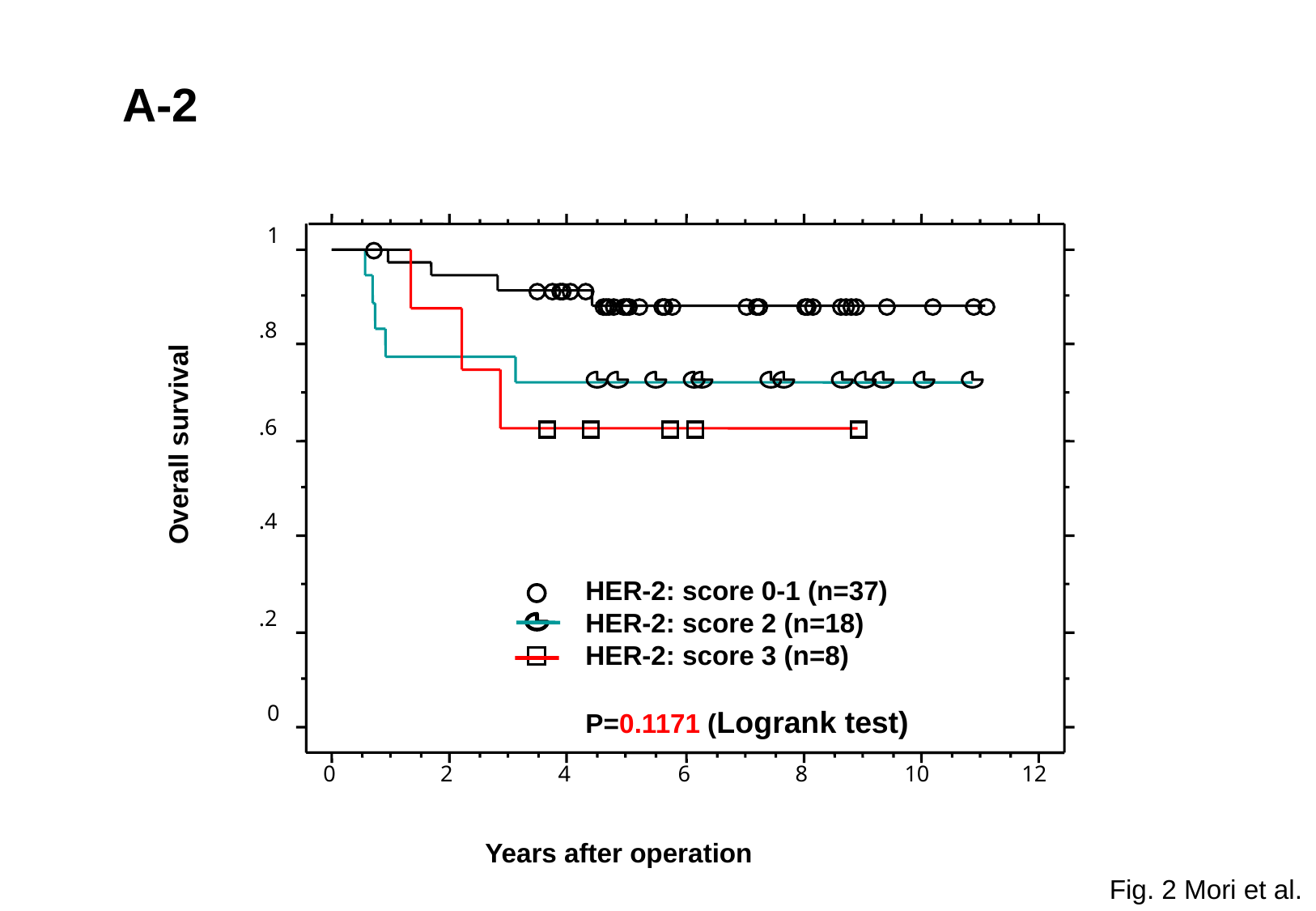

1
.8
.6
.4
.2
0
0
2
4
6
8
10
12
A-2
Overall survival
HER-2: score 0-1 (n=37)
HER-2: score 2 (n=18)
HER-2: score 3 (n=8)
P=0.1171 (Logrank test)
Years after operation
Fig. 2 Mori et al.

## Slide 10
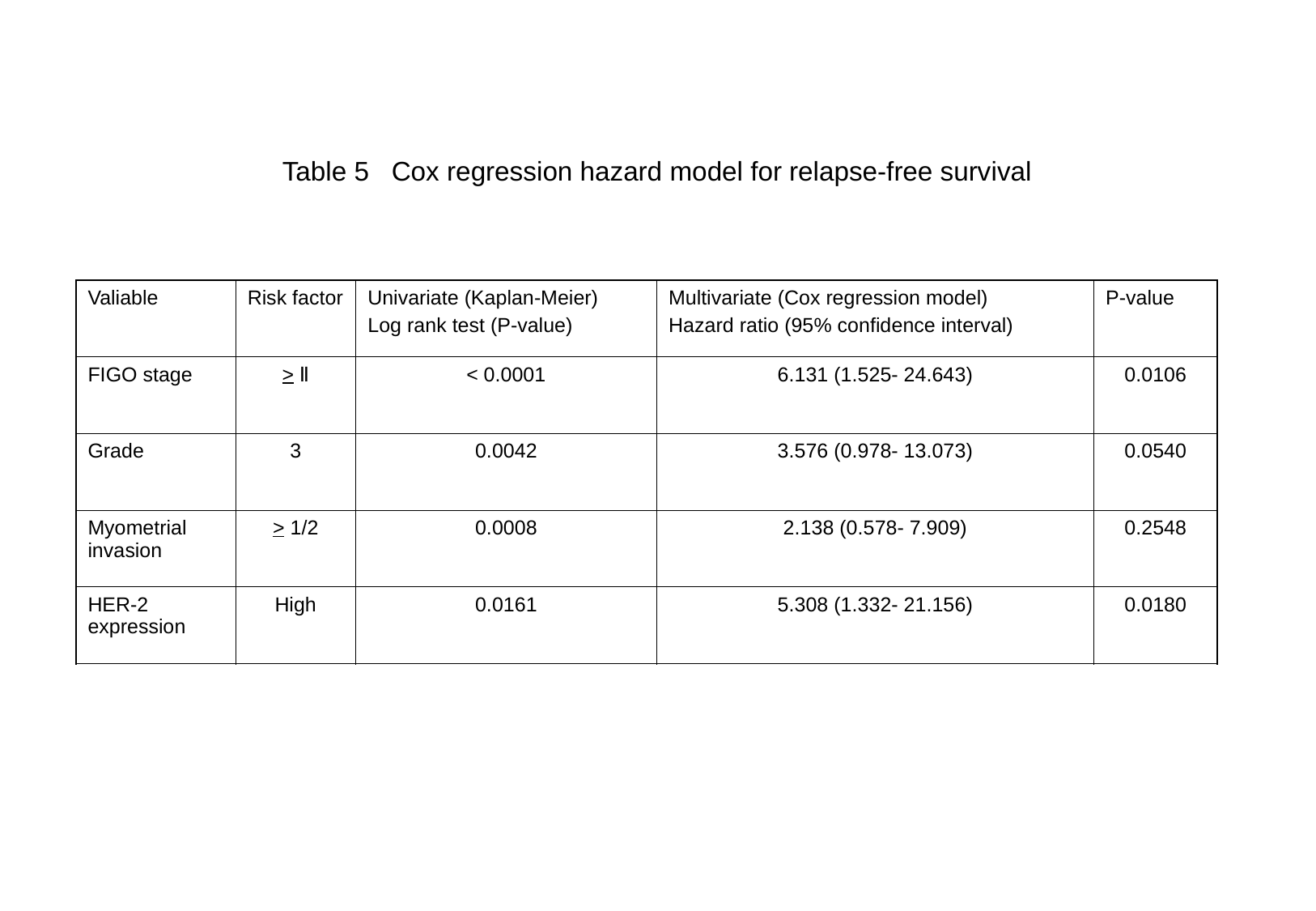

Table 5 Cox regression hazard model for relapse-free survival
| Valiable | Risk factor | Univariate (Kaplan-Meier) Log rank test (P-value) | Multivariate (Cox regression model) Hazard ratio (95% confidence interval) | P-value |
| --- | --- | --- | --- | --- |
| FIGO stage | > Ⅱ | < 0.0001 | 6.131 (1.525- 24.643) | 0.0106 |
| Grade | 3 | 0.0042 | 3.576 (0.978- 13.073) | 0.0540 |
| Myometrial invasion | > 1/2 | 0.0008 | 2.138 (0.578- 7.909) | 0.2548 |
| HER-2 expression | High | 0.0161 | 5.308 (1.332- 21.156) | 0.0180 |

## Slide 11
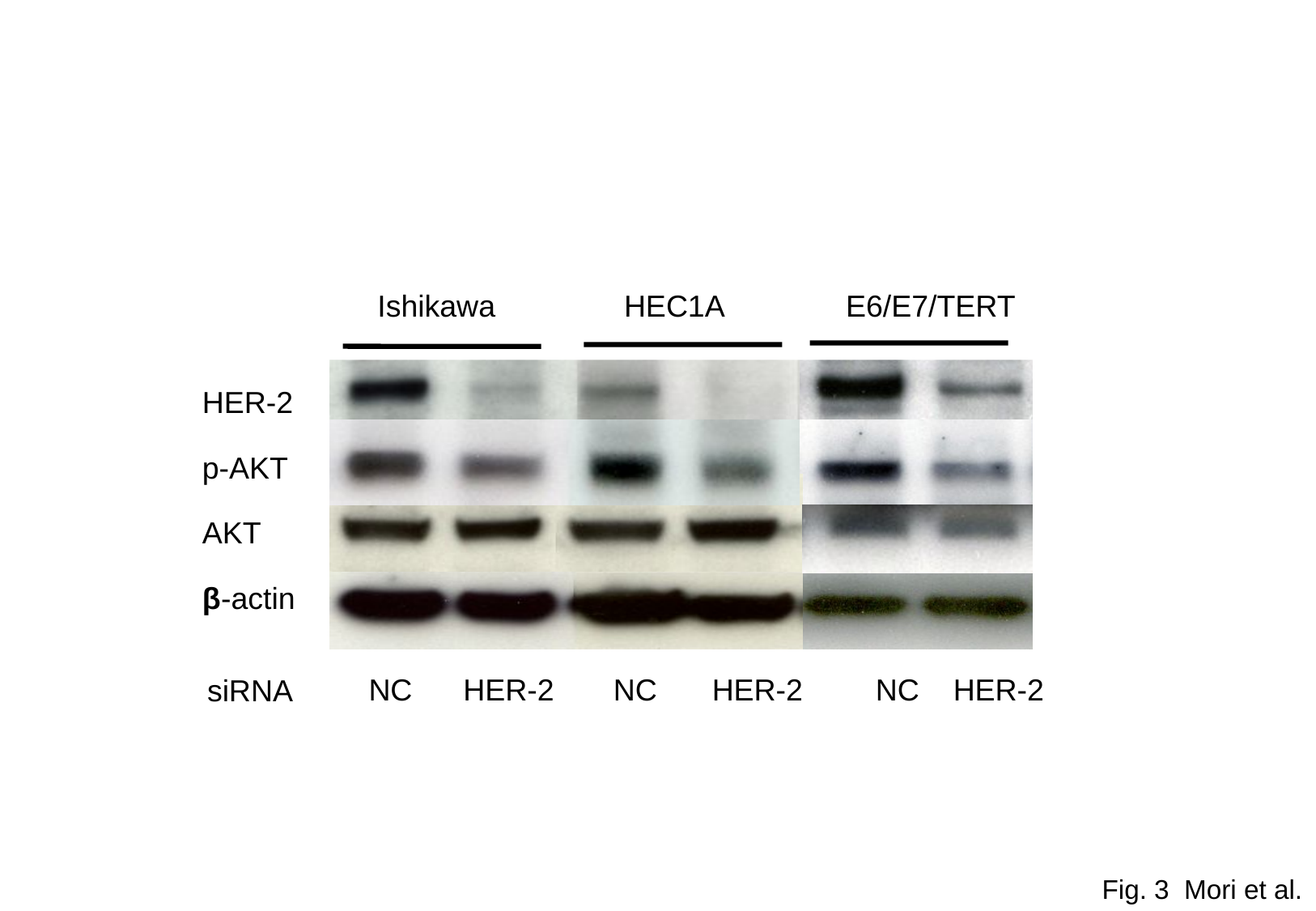

Ishikawa	　 HEC1A 　　 E6/E7/TERT
HER-2
p-AKT
AKT
β-actin
 NC HER-2 NC　 HER-2 　 NC HER-2
siRNA
Fig. 3 Mori et al.

## Slide 12
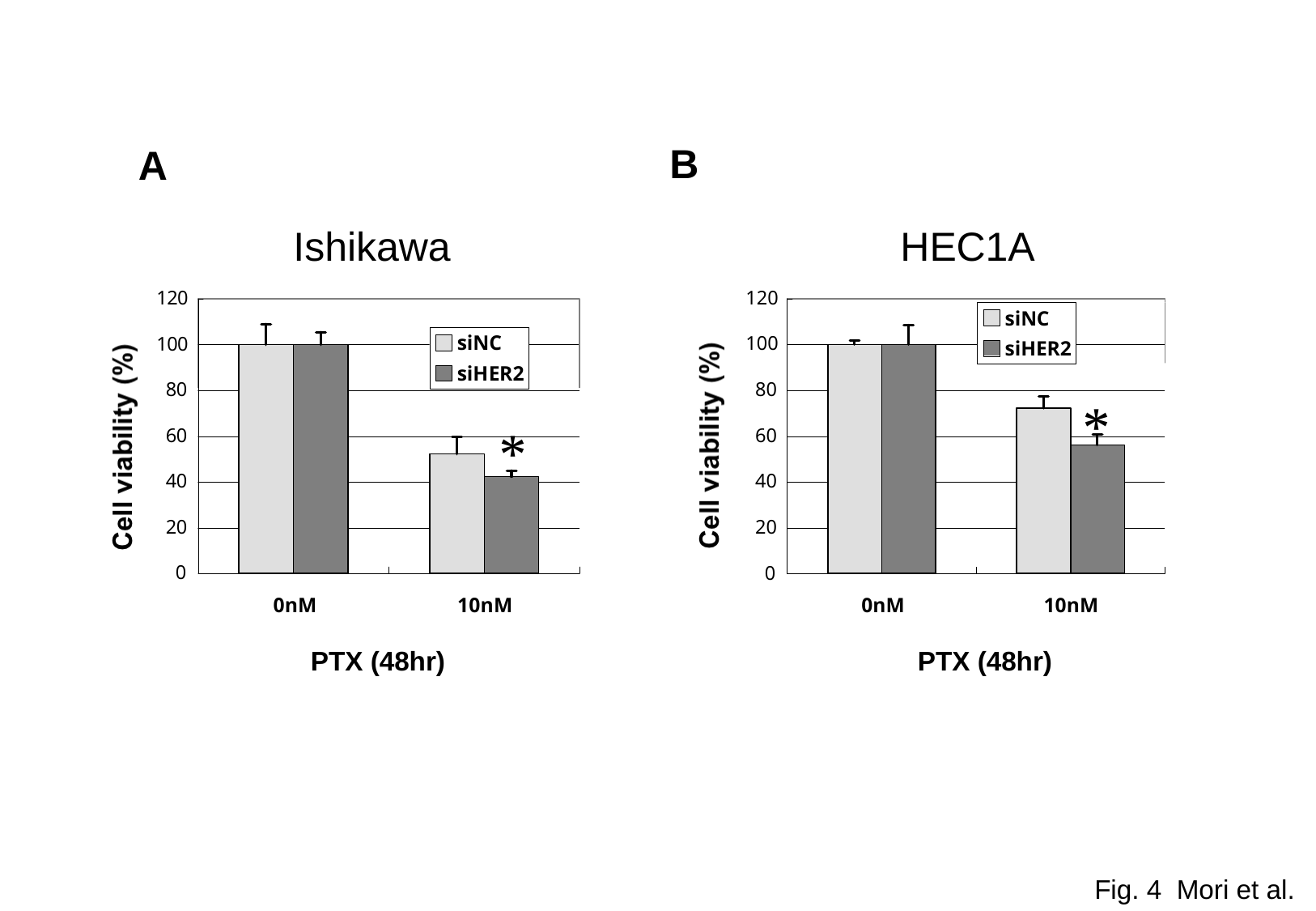

B
A
Ishikawa				HEC1A
*
*
*
*
PTX (48hr) 				PTX (48hr)
Fig. 4 Mori et al.

## Slide 13
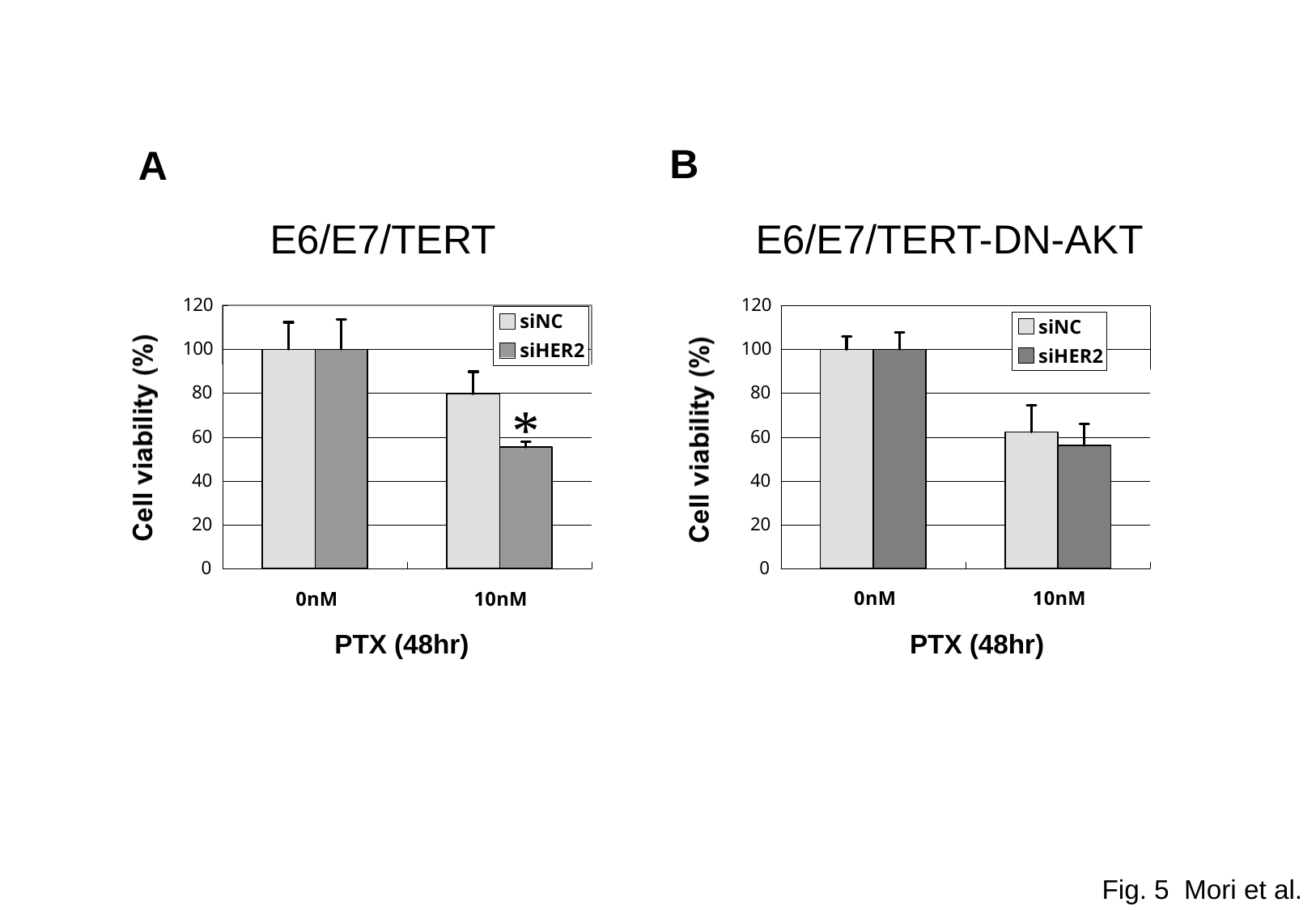

B
A
E6/E7/TERT			E6/E7/TERT-DN-AKT
*
PTX (48hr) 			 PTX (48hr)
Fig. 5 Mori et al.

## Slide 14
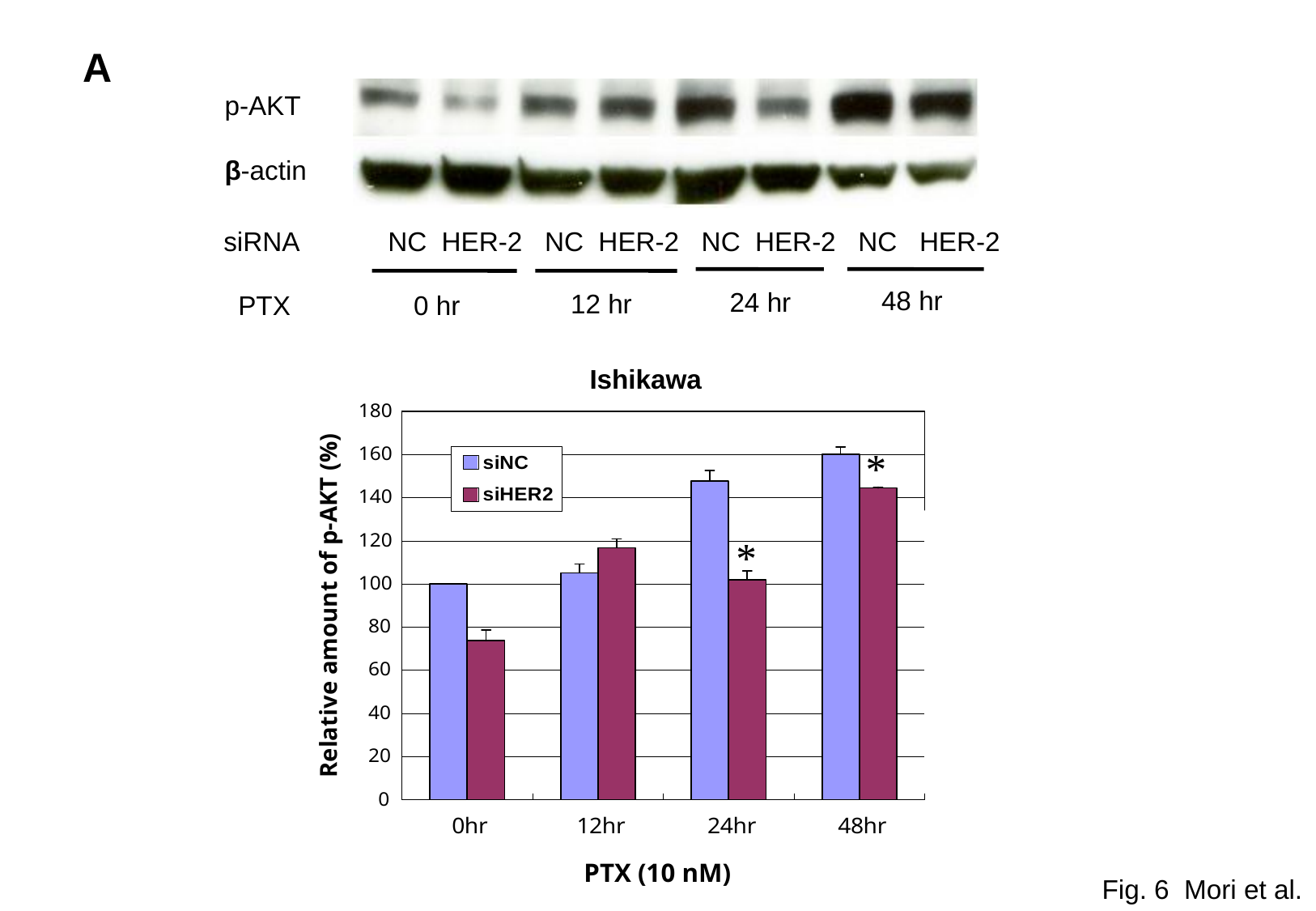

A
p-AKT
β-actin
 siRNA	　 NC HER-2 NC HER-2 NC HER-2 NC HER-2
48 hr
24 hr
12 hr
PTX
0 hr
Ishikawa
*
*
Relative amount of p-AKT (%)
PTX (10 nM)
Fig. 6 Mori et al.

## Slide 15
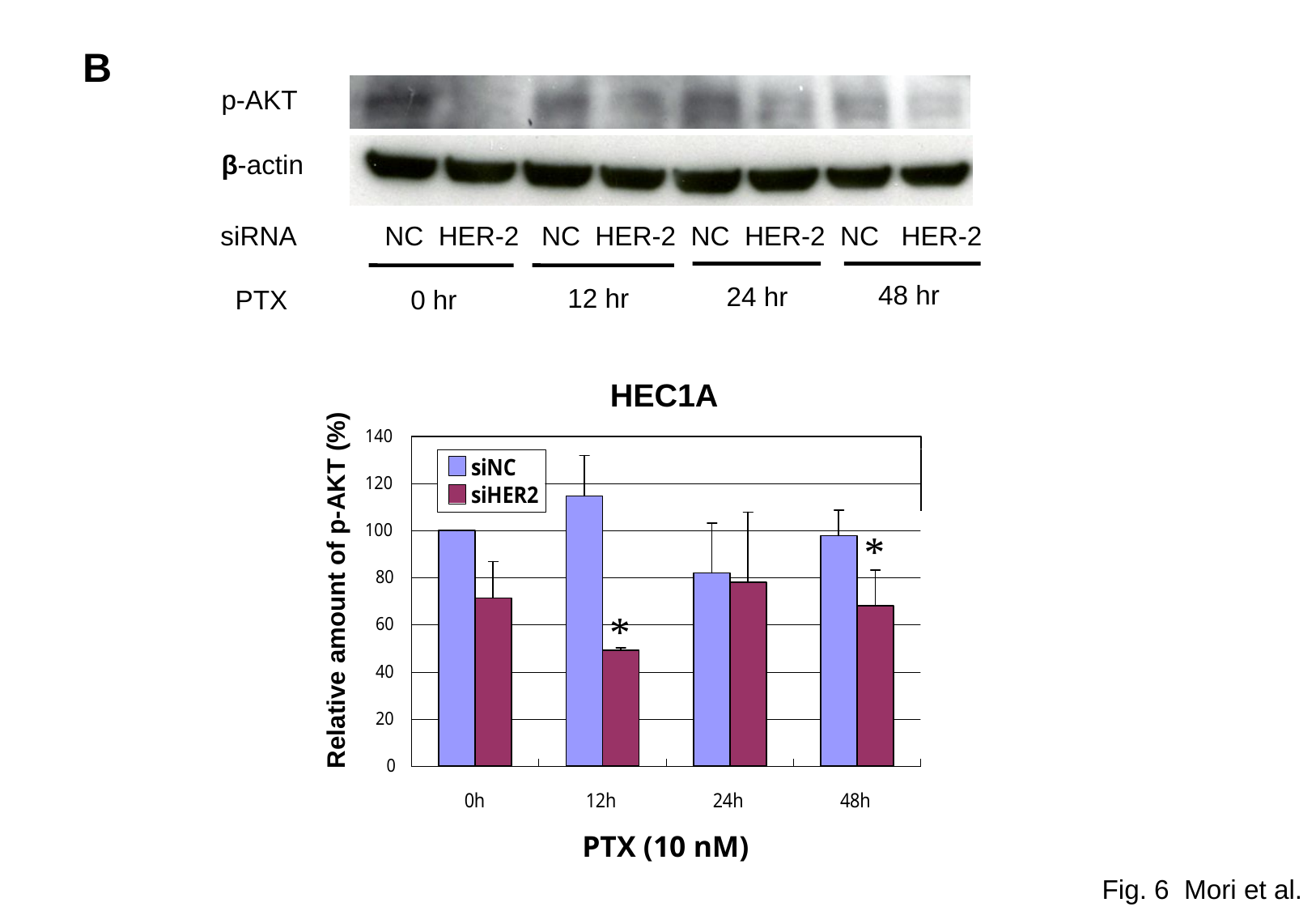

B
p-AKT
β-actin
 siRNA	　 NC HER-2 NC HER-2 NC HER-2 NC HER-2
48 hr
24 hr
12 hr
PTX
0 hr
HEC1A
*
Relative amount of p-AKT (%)
*
 PTX (10 nM)
Fig. 6 Mori et al.

## Slide 16
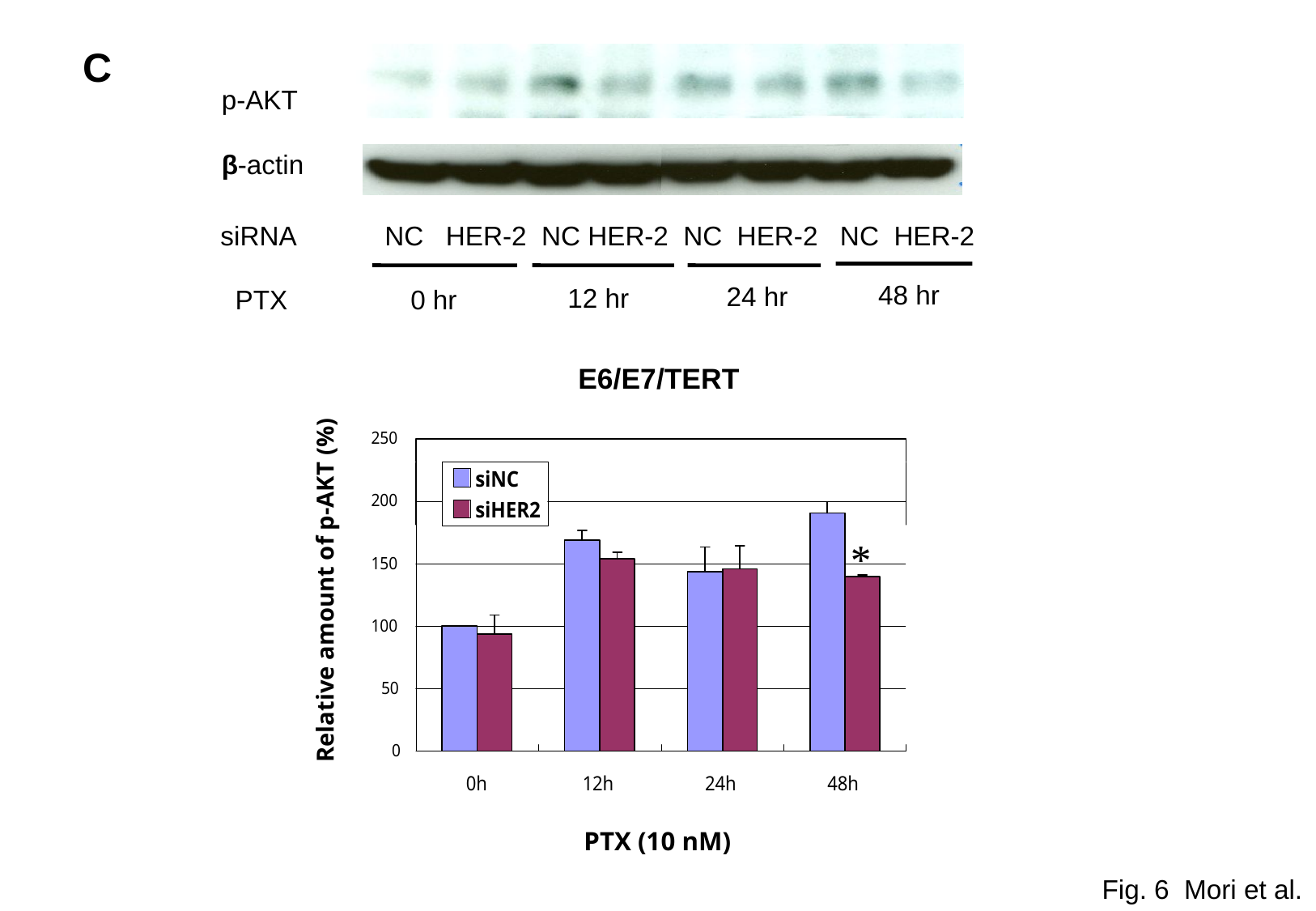

C
p-AKT
β-actin
 siRNA	　 NC HER-2 NC HER-2 NC HER-2 NC HER-2
48 hr
24 hr
12 hr
PTX
0 hr
48 hr
24 hr
12 hr
0 hr
E6/E7/TERT
E6/E7/TERT
E6/E7/TERT
*
*
Relative amount of p-AKT (%)
PTX (10 nM)
Fig. 6 Mori et al.

## Slide 17
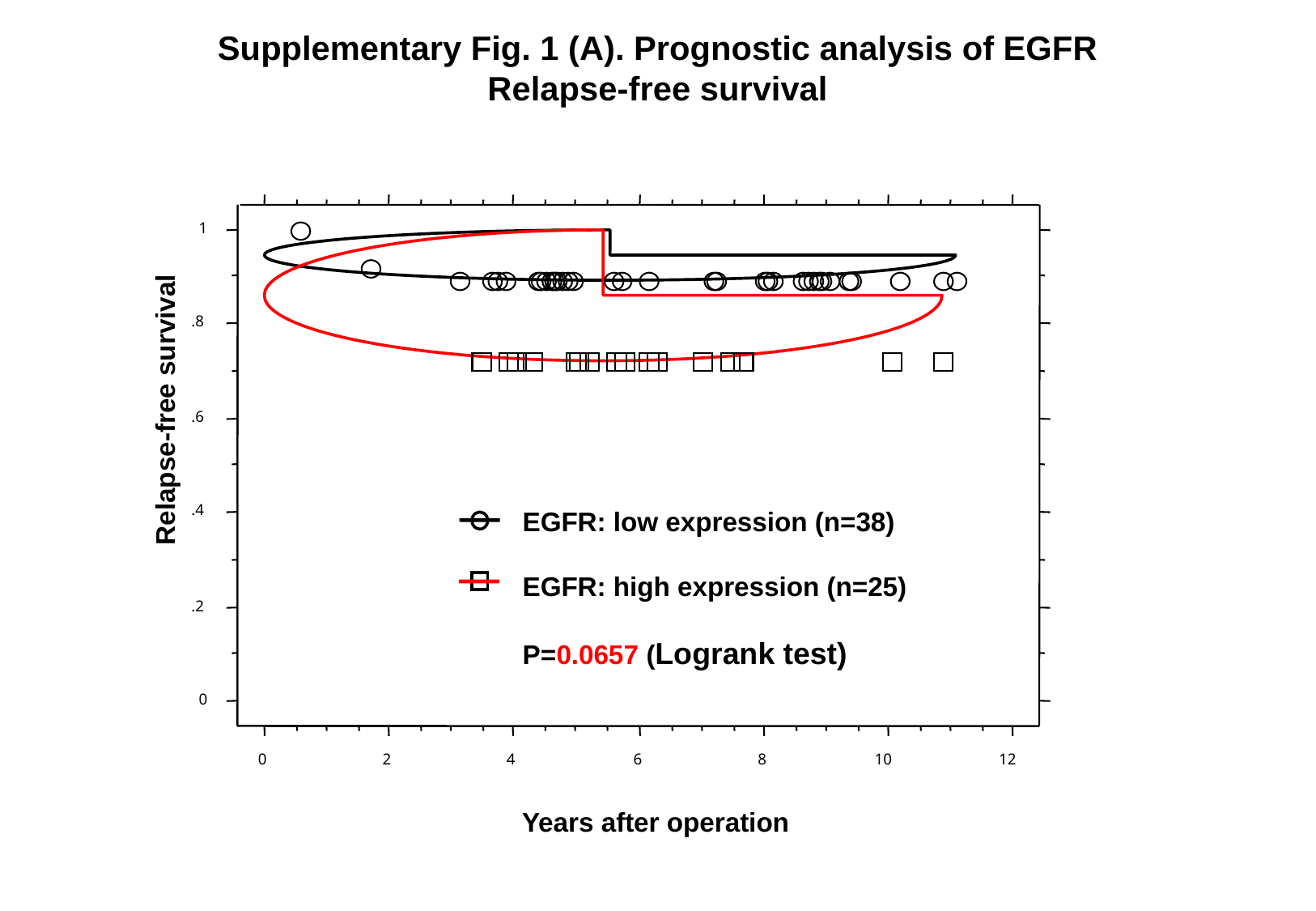

Supplementary Fig. 1 (A). Prognostic analysis of EGFR
Relapse-free survival
1
.8
.6
.4
.2
0
0
2
4
6
8
10
12
Relapse-free survival
EGFR: low expression (n=38)
EGFR: high expression (n=25)
P=0.0657 (Logrank test)
Years after operation

## Slide 18
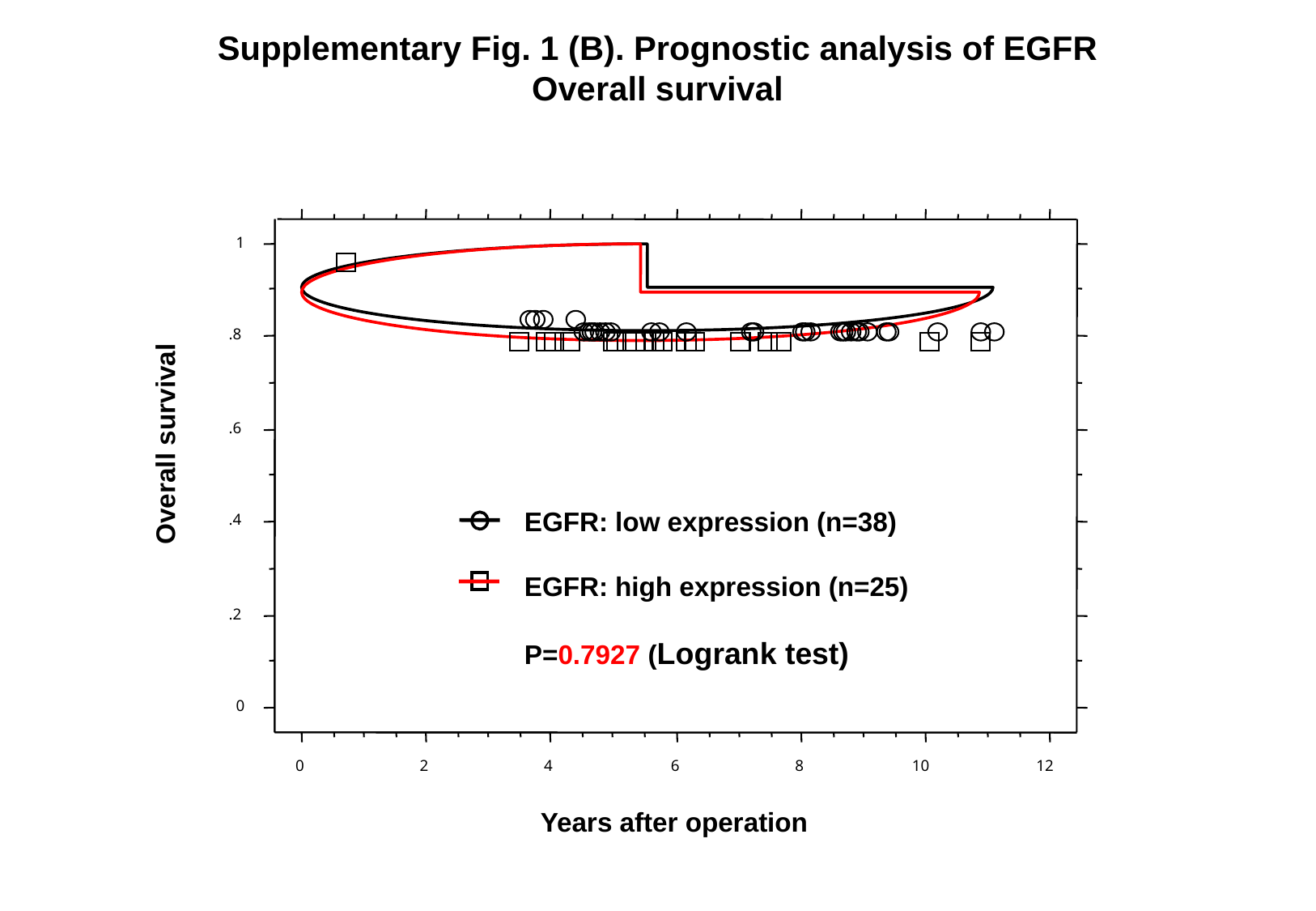

Supplementary Fig. 1 (B). Prognostic analysis of EGFR
Overall survival
1
.8
.6
.4
.2
0
0
2
4
6
8
10
12
Overall survival
EGFR: low expression (n=38)
EGFR: high expression (n=25)
P=0.7927 (Logrank test)
Years after operation

## Slide 19
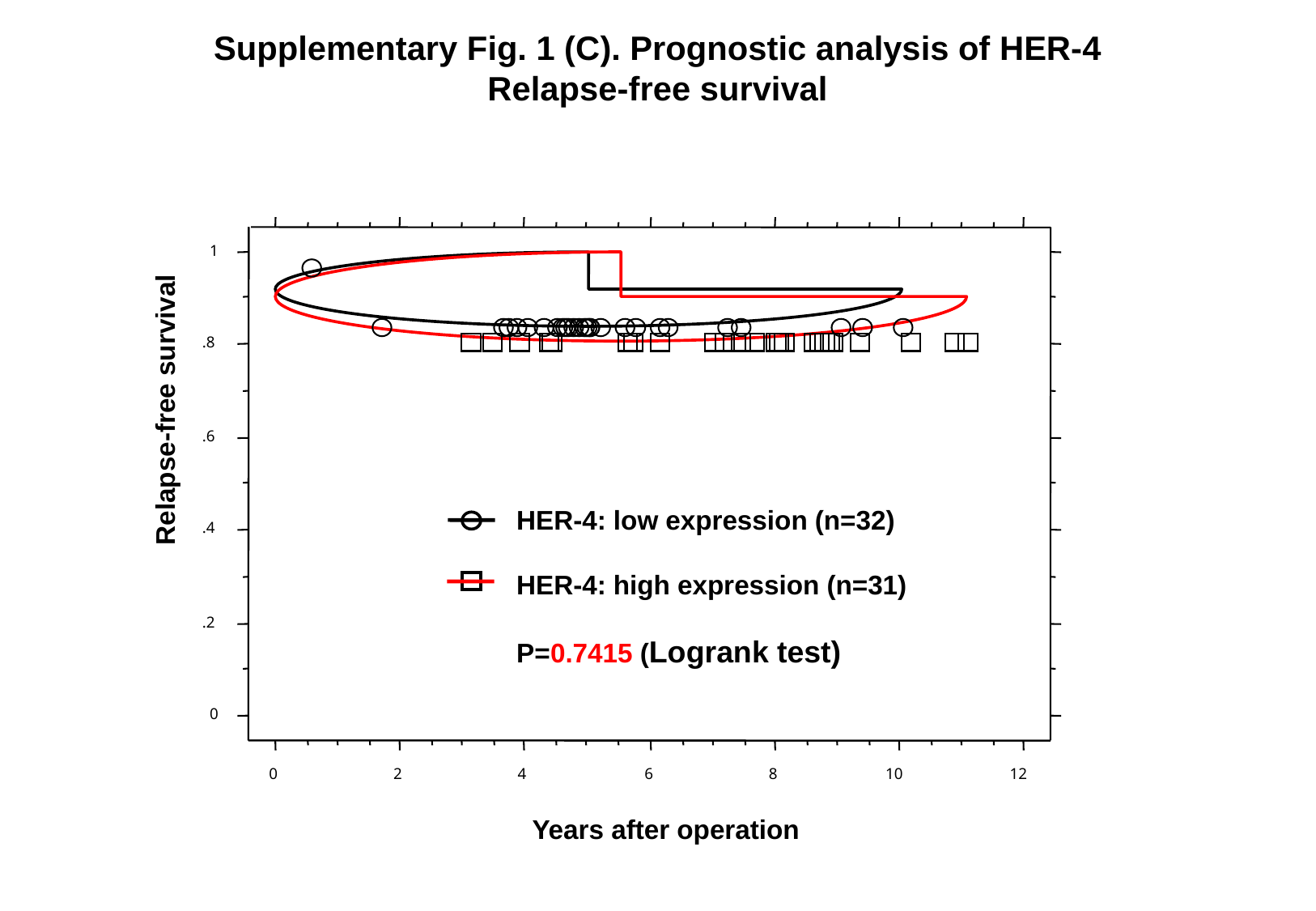

Supplementary Fig. 1 (C). Prognostic analysis of HER-4
Relapse-free survival
1
.8
.6
.4
.2
0
0
2
4
6
8
10
12
Relapse-free survival
HER-4: low expression (n=32)
HER-4: high expression (n=31)
P=0.7415 (Logrank test)
Years after operation

## Slide 20
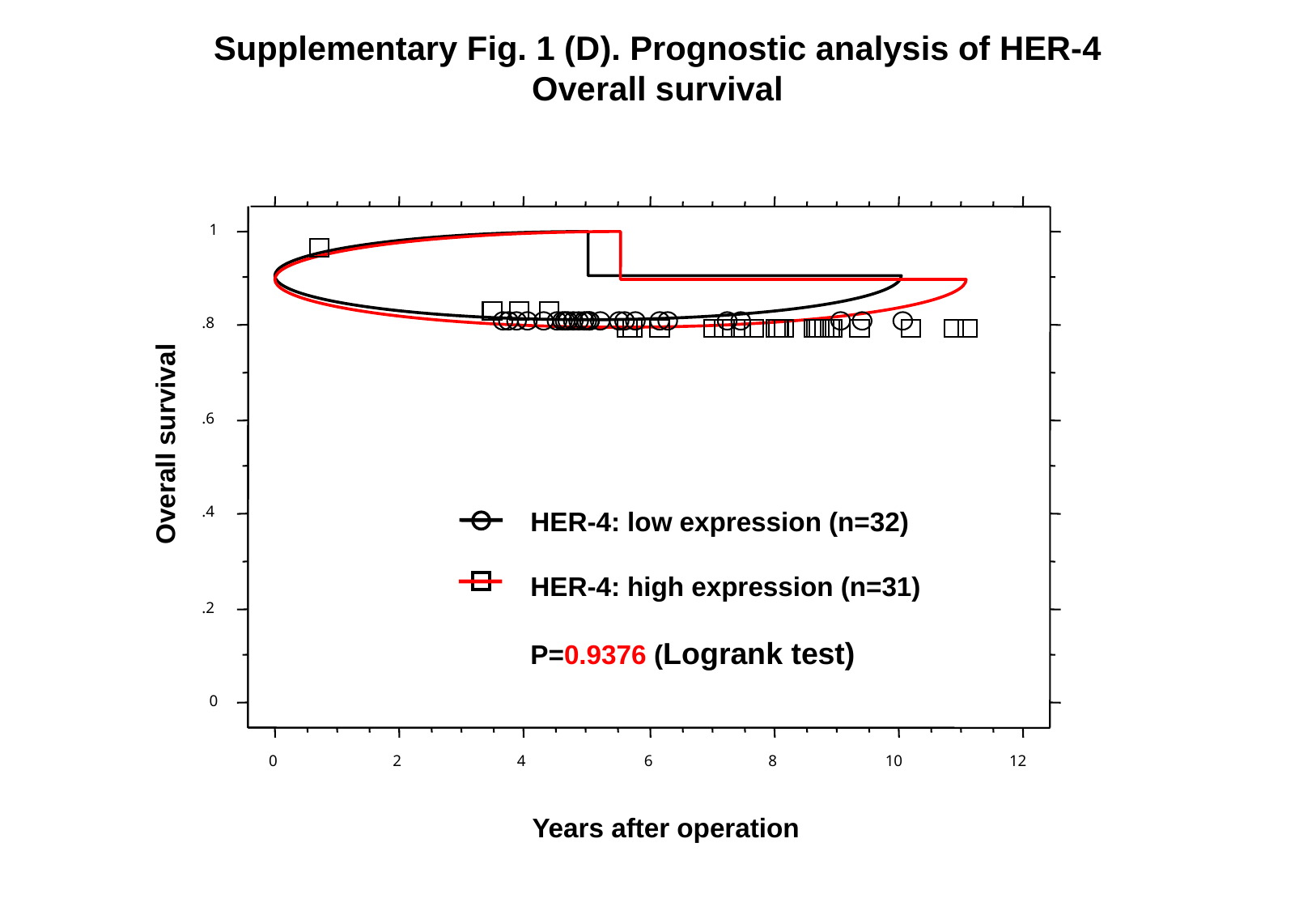

Supplementary Fig. 1 (D). Prognostic analysis of HER-4
Overall survival
1
.8
.6
.4
.2
0
0
2
4
6
8
10
12
Overall survival
HER-4: low expression (n=32)
HER-4: high expression (n=31)
P=0.9376 (Logrank test)
Years after operation
