# Supplementary material for: Expression of HER-2 affects patient survival and paclitaxel sensitivity in endometrial cancer
Source: Br J Cancer. 2010 Jul 27;103(6):889–98. doi: 10.1038/sj.bjc.6605805 (PMC2966616; doi:10.1038/sj.bjc.6605805)
Supplement: Supplementary Figure Legend [file 6605805x2.doc]

**Supplementary Fig.1 Kaplan-Meier survival curves of 63 patients with endometrial cancer in relation to EGFR or HER-4 expression.**
